# A single-cell and single-nucleus RNA-Seq toolbox for fresh and frozen human tumors

**DOI:** 10.1038/s41591-020-0844-1

**Published:** 2020-05-11

**Authors:** Michal Slyper, Caroline B. M. Porter, Orr Ashenberg, Julia Waldman, Eugene Drokhlyansky, Isaac Wakiro, Christopher Smillie, Gabriela Smith-Rosario, Jingyi Wu, Danielle Dionne, Sébastien Vigneau, Judit Jané-Valbuena, Timothy L. Tickle, Sara Napolitano, Mei-Ju Su, Anand G. Patel, Asa Karlstrom, Simon Gritsch, Masashi Nomura, Avinash Waghray, Satyen H. Gohil, Alexander M. Tsankov, Livnat Jerby-Arnon, Ofir Cohen, Johanna Klughammer, Yanay Rosen, Joshua Gould, Lan Nguyen, Matan Hofree, Peter J. Tramontozzi, Bo Li, Catherine J. Wu, Benjamin Izar, Rizwan Haq, F. Stephen Hodi, Charles H. Yoon, Aaron N. Hata, Suzanne J. Baker, Mario L. Suvà, Raphael Bueno, Elizabeth H. Stover, Michael R. Clay, Michael A. Dyer, Natalie B. Collins, Ursula A. Matulonis, Nikhil Wagle, Bruce E. Johnson, Asaf Rotem, Orit Rozenblatt-Rosen, Aviv Regev

**Affiliations:** 1 Klarman Cell Observatory, Broad Institute of Harvard and MIT, Cambridge, MA USA; 2 Broad Institute of Harvard and MIT, Cambridge, MA USA; 3 Department of Medical Oncology, Dana-Farber Cancer Institute, Boston, MA USA; 4 Center for Cancer Precision Medicine of Dana-Farber Cancer Institute, Boston, MA USA; 5 Department of Developmental Neurobiology, St Jude Children’s Research Hospital, Memphis, TN USA; 6 Department of Oncology, St Jude Children’s Research Hospital, Memphis, TN USA; 7 Department of Pathology, Massachusetts General Hospital and Harvard Medical School, Boston, MA USA; 8 Center for Cancer Research, Massachusetts General Hospital and Harvard Medical School, Boston, MA USA; 9 Center for Regenerative Medicine, Massachusetts General Hospital, Boston, MA USA; 10 Division of Thoracic Surgery, Brigham and Women’s Hospital, Boston, MA USA; 11 Center for Immunology and Inflammatory Diseases, Division of Rheumatology, Allergy, and Immunology, Massachusetts General Hospital and Harvard Medical School, Boston, MA USA; 12 Department of Internal Medicine, Brigham and Women’s Hospital, Boston, MA USA; 13 Harvard Medical School, Boston, MA USA; 14 Laboratory for Systems Pharmacology, Harvard Medical School, Boston, MA USA; 15 Center for Immunology and Virology, Dana-Farber Cancer Institute, Boston, MA USA; 16 Ludwig Center for Cancer Research at Harvard, Boston, MA USA; 17 Melanoma Disease Center, Dana-Farber Cancer Institute, Boston, MA USA; 18 Department of Surgical Oncology, Brigham and Women’s Hospital, Boston, MA USA; 19 Department of Medicine, Massachusetts General Hospital and Harvard Medical School, Boston, MA USA; 20 Department of Pathology, St Jude Children’s Research Hospital, Memphis, TN USA; 21 Howard Hughes Medical Institute, Chevy Chase, MD USA; 22 Department of Pediatric Oncology, Dana-Farber Cancer Institute, Boston, MA USA; 23 Division of Pediatric Hematology and Oncology, Boston Children’s Hospital, Boston, MA USA; 24 Koch Institute for Integrative Cancer Research, Department of Biology, Massachusetts Institute of Technology, Cambridge, MA USA; 25 Present Address: Icahn School of Medicine at Mount Sinai, New York, NY USA; 26 Present Address: Columbia Center for Translational Immunology and Division of Hematology and Oncology, Columbia University Medical Center, New York, NY USA

**Keywords:** Gene expression analysis, Cancer, Computational biology and bioinformatics, Biological techniques, Genomic analysis

## Abstract

Single-cell genomics is essential to chart tumor ecosystems. Although single-cell RNA-Seq (scRNA-Seq) profiles RNA from cells dissociated from fresh tumors, single-nucleus RNA-Seq (snRNA-Seq) is needed to profile frozen or hard-to-dissociate tumors. Each requires customization to different tissue and tumor types, posing a barrier to adoption. Here, we have developed a systematic toolbox for profiling fresh and frozen clinical tumor samples using scRNA-Seq and snRNA-Seq, respectively. We analyzed 216,490 cells and nuclei from 40 samples across 23 specimens spanning eight tumor types of varying tissue and sample characteristics. We evaluated protocols by cell and nucleus quality, recovery rate and cellular composition. scRNA-Seq and snRNA-Seq from matched samples recovered the same cell types, but at different proportions. Our work provides guidance for studies in a broad range of tumors, including criteria for testing and selecting methods from the toolbox for other tumors, thus paving the way for charting tumor atlases.

## Abstract (web summary)

A set of ready-to-use tools for profiling fresh and frozen clinical tumor samples using scRNA-Seq and snRNA-Seq facilitates the implementation of single-cell technologies in clinical settings and the construction of single-cell tumor atlases.

## Main

Tumors encompass complex cellular ecosystems of malignant and non-malignant cells, whose diversity and interactions affect cancer progression and drug response and resistance. Recent advances in single-cell genomics, especially single-cell RNA-Seq (scRNA-Seq), have transformed our ability to analyze tumors, revealing cell types, states, genetic diversity and interactions in the complex tumor ecosystem^1–6^. Single-cell analysis of tumors is rapidly expanding, including the launch of a Human Tumor Atlas Network (HTAPP) as part of the Cancer Moonshot^7^.

Successful scRNA-Seq of clinical tumor specimens poses several challenges. First, it requires quick dissociation tailored to the tumor type, and involves enzymatic digestion, which can lead to loss of sensitive cells or changes in gene expression. Moreover, obtaining fresh tissue is time-sensitive and requires tight coordination between tissue acquisition and processing teams, posing a challenge in clinical settings. Conversely, single-nucleus RNA-Seq (snRNA-Seq) allows profiling of single nuclei isolated from frozen tissues, decoupling tissue acquisition from immediate sample processing. snRNA-Seq can also handle samples that cannot be successfully dissociated even when fresh, due to size or cell fragility^8,9^, as well as multiplexed analysis of longitudinal samples from the same individual^10^. However, nuclei have lower amounts of mRNA compared to cells and are more challenging to enrich or deplete for specific cell types of interest. Both scRNA-Seq and snRNA-Seq pose experimental challenges when applied to different tumor types, due to the distinct cellular composition and extracellular matrix (ECM) in different tumors, and thus each assay requires dedicated customizations^11^.

**Fig. 1:**
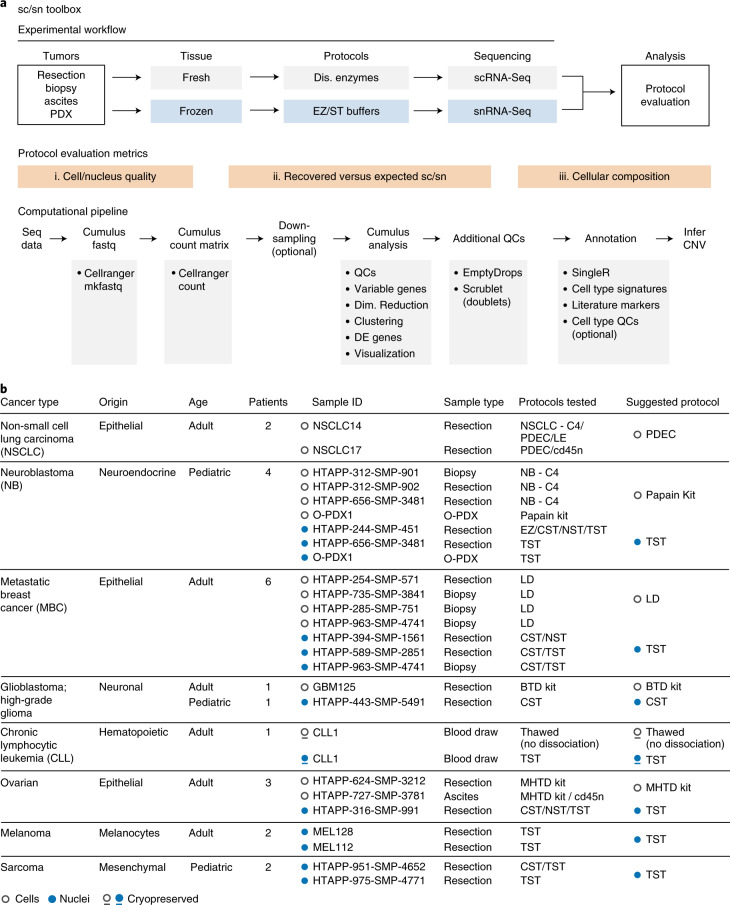
Study and toolbox overview. **a**, sc/snRNA-Seq workflow, experimental and computational pipelines, and protocol selection criteria. **b**, Tumor types and samples processed in the study. Tested and selected protocols for fresh (white circles, cells), frozen (blue circles, nuclei) and cryopreserved (underlined circles, cells (white) and nuclei (blue)) are indicated. O-PDX, orthotopic patient-derived xenograft; EZ, EZPrep; ST, salts and Tris; QC, quality control; DE, differentially expressed; InferCNV, Infer Copy Number Variation, a method for detecting copy number abberations; C4, collagenase 4 and DNase I; PDEC, pronase, dispase, elastase, collagenases A and 4 and DNase I; LE, Liberase TM, elastase and DNase I; EZ, EZPrep; NST, Nonidet P40 with salts and Tris; CST, CHAPS with salts and Tris; TST, Tween with salts and Tris; LD, Liberase TM and DNase I; BTD, brain tumor dissociation; MHTD, Miltenyi Biotec human tumor dissociation.

To address these challenges, we developed a systematic toolbox for fresh and frozen tumor processing using scRNA-Seq and snRNA-Seq, respectively (Fig. 1a). The toolbox contains our experimental workflow and methods, computational pipelines and evaluation metrics. To generalize across tumor and sample types, we tested eight tumor types with different tissue characteristics (Fig. 1b), including comparisons of matched fresh and frozen preparations from the same tumor specimen. Our work provides direct recommended protocols for multiple tumor types, decision trees that allow researchers to choose the most suitable protocol for their research goals, and guidelines on how to customize protocols for new tumor and specimen types.

## Results

### A systematic study to develop an sc/snRNA-Seq toolbox

To develop a toolbox of customized protocols for sc/snRNA-Seq of tumors, we studied eight tumor types with different tissue characteristics (Fig. 1). The tumor types span the following characteristics: different cells of origin (for example, epithelial, neuronal), solid and non-solid, patient ages and transitions (for example, primary, metastatic). We tested varying tissue and sample characteristics including resection, biopsy, ascites and orthotopic patient-derived xenograft (O-PDX). We included samples from non-small cell lung carcinoma (NSCLC), metastatic breast cancer (MBC), ovarian cancer, neuroblastoma, glioblastoma (GBM), pediatric high-grade glioma, chronic lymphocytic leukemia (CLL), pediatric sarcoma and melanoma (Fig. 1b). In total, we analyzed 216,490 cells and nuclei across 23 tumors, from 22 patients spanning 40 sample preparations. We provide a comprehensive analysis summary for each sample tested in a dedicated website (https://tumor-toolbox.broadinstitute.org).

### Experimental and computational QCs assess quality and composition

We evaluated and compared protocols based on (1) cell/nucleus quality; (2) number of recovered versus expected cells/nuclei; (3) cellular composition (Fig. 1a). For ‘cell/nucleus quality’, we considered both experimental and computational metrics. Experimentally, we measured cell viability (for scRNA-Seq), the extent of doublets or aggregates in the cell/nucleus suspension and cDNA quality recovered after whole transcriptome amplification. Computationally, we evaluated the percent of reads mapping to the transcriptome, genome and intergenic regions, the number of cells/nuclei exceeding a minimal number of genes and unique transcripts (reflected by unique molecular identifiers, UMIs), the number of reads, transcripts (UMIs) and genes detected per cell/nucleus and the percent of UMIs from mitochondrial genes (see Methods). To compare protocols, when there was a notable difference in sequencing saturation or total reads across samples, we also downsampled reads to equal numbers across samples and then re-estimated and compared their QC metrics. For ‘number of recovered versus expected cells/nuclei’, we evaluated the proportion of droplets scored as likely empty (that is, containing only ambient RNA rather than the RNA from an encapsulated cell^12^), and the proportion of doublets^13^ (see Methods). Because the algorithm for scoring empty droplets was developed for cells, we did not use it to evaluate snRNA-Seq. Finally, for ‘cellular composition’, we considered the diversity of cell types captured, the proportion of cells/nuclei recovered from each subset and the copy number aberration (CNA) pattern classes that are recovered in malignant cells (see Methods). We considered it a virtue if a protocol recovers a larger diversity of cell types, because this facilitates comprehensive studies. However, capture of diverse cell types may not always be a researcher’s desired goal, nor would it always be the most accurate representation of a tumor’s composition (see Discussion). We annotated the malignant cells based on the presence of CNAs (when detectable) and the cell type signature they most closely resembled (see Methods). We conducted initial data analysis using Cumulus, a cloud-based data analysis framework^14^ (see Methods and Fig. 1a), and developed a dedicated pipeline for additional quality control, tumor sample characterization and protocol comparison.

### Customization of workflows and dissociation protocols for scRNA-Seq of fresh tumors

**Fig. 2:**
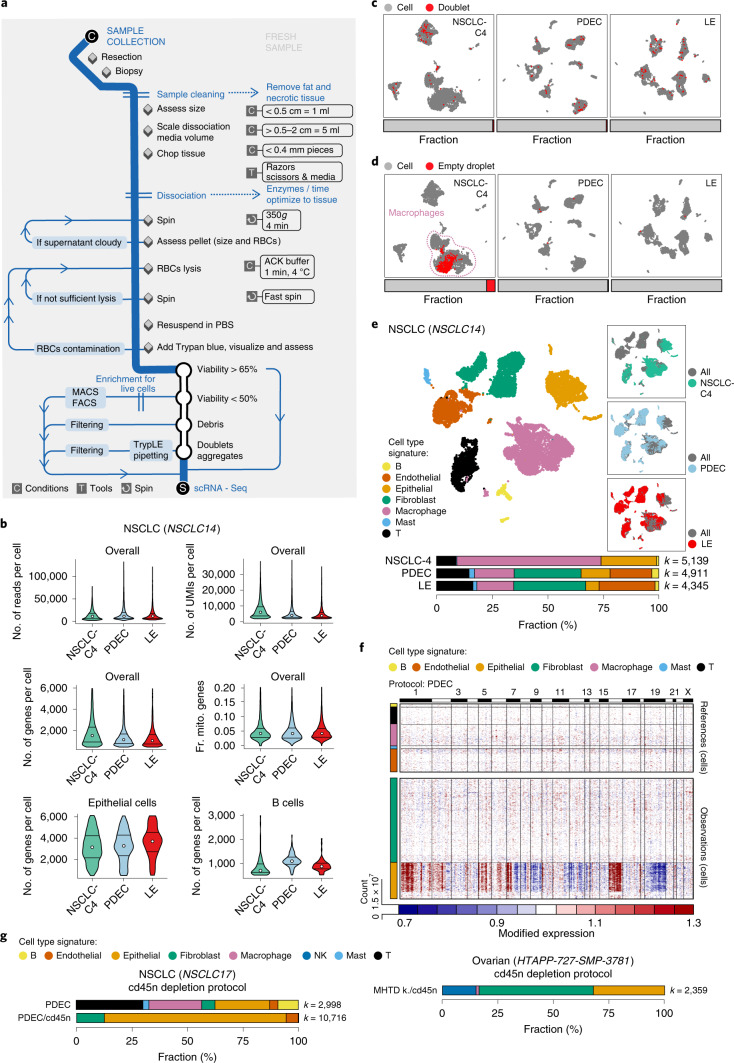
Fresh tumor processing and protocol selection for scRNA-Seq. **a**, Flow chart recommended for collection and processing of fresh tumor samples. **b**–**f**, Comparison of three dissociation protocols applied to one NSCLC sample. **b**, Protocol performance varies across cell types. Top and middle: distribution (median and first and third quartiles) of the number of reads per cell, the number of UMIs per cell, the number of genes per cell and fraction of UMIs per cell mapping to mitochondrial genes (Fr. mito. genes) (*y* axes) in each protocol (*x* axis) across the entire dataset. Bottom: distribution (median and first and third quartiles) of the number of genes per cell (*y* axis) only in epithelial cells (left) or in B cells (right). **c**, The protocols detect similar numbers of doublets. Uniform manifold approximation and projection (UMAP) embedding of single cell profiles (dots) for each protocol, colored by assignment as single cell (gray) or doublet (red). Horizontal bars (bottom): fraction of single (gray) and doublet (red) cells. **d**, The protocols vary in the number of empty drops. UMAP embedding of single cell profiles (dots) for each protocol, colored by assignment as cell (gray) or empty drop (red). Horizontal bars (bottom): fraction of assigned cells (gray) and empty drops (red). **e**, The protocols vary in the diversity of cell types captured. UMAP embedding of single cell profiles (dots) from all three protocols, colored by assigned cell subset signature (left) or by protocol (right). Bottom: proportion of cells in each subset in each of the three protocols; *k*, number of cells passing QC. **f**, Inferred CNA profiles. Chromosomal amplification (red) and deletion (blue) are inferred in each chromosomal position (columns) across the single cells (rows) using the PDEC protocol. Top: reference cells not expected to contain CNAs in this tumor. Bottom: cells tested for CNAs relative to the reference cells. Color bar: assigned cell type signature for each cell. **g**, Successful depletion of CD45^+^ cells. The proportion of cells in each subset without and with CD45^+^ depletion in NSCLCs (top) and ovarian ascites (bottom) samples is shown; *k*, number of cells passing QC. *n* = 1 sample per protocol. The numbers of cells (*k*) are indicated in **e** and **g**. Numbers of epithelial cells from NSCLC-C4, PDEC and LE are *k* = 1,284, 641 and 260, respectively, and the number of B cells is *k* = 100, 121 and 78, respectively. ACK, ammonium-chloride-potassium.

We customized successful protocols for specimen acquisition and dissociation for scRNA-Seq across five types of fresh tumor (NSCLC, ovarian cancer, MBC, neuroblastoma, GBM and cryopreserved CLL (Fig. 1b)). We constructed workflows that minimize the time interval between removal of the sample from the patient in a clinical setting and its dissociation into cells, to maximize cell viability and preservation of RNA profiles. We determined dissociation conditions for each tumor type and constructed specific steps as a decision tree to adjust for differences between clinical samples (for example, size, presence of red blood cells (RBCs); Fig. 2a and Methods). To choose the best performing dissociation method, when possible, we apportioned large tumor specimens into smaller pieces (~0.5–2 cm), dissociating each piece following a different protocol. When specimen size was limiting (for example, biopsies), optimization spanned multiple samples. We subjected the samples that yielded highly viable single cell suspensions to droplet-based scRNA-Seq (see Methods), to allow sampling of larger number of cells for calculation of QC metrics. We tested protocols several times to confirm similar performance trends.

For dissociation, we selected enzymatic mixtures for processing fresh tissues based on the specific characteristics of each tumor type, such as cell type composition and ECM components, literature review and experience from processing similar human or mouse tissues. For example, to break down collagen fibers in breast cancer^15,16^ we used Liberase TM (see Methods), whereas to break down ECM in GBM^17^ we used papain (cysteine protease). We also included DNase I to digest DNA released from dead cells to decrease viscosity in all dissociation mixtures. In the following, we recommend those methods that broke down the ECM and cell-to-cell adhesions sufficiently, while minimizing processing time, maintaining high viability and supporting cell type diversity in the sample.

### Cell-type-specific and cell-composition QCs are important in protocol evaluation

**Fig. 3:**
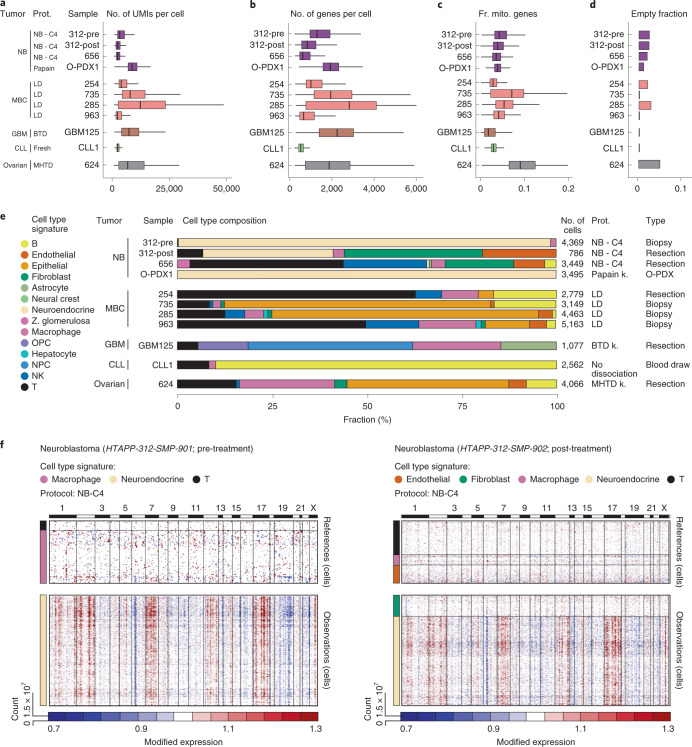
scRNA-Seq protocol comparison across tumor types. **a**–**d**, QC metrics. The number of UMIs per cell (**a**), number of genes per cell (**b**), fraction of UMIs per cell mapping to mitochondrial genes (**c**) and fraction of empty drops (**d**) (*x* axes) for each sample (*y* axis). Median and first and third quartiles are shown in **a**–**c**. **e**, Cell type composition. Proportion of cells assigned to each cell type signature (color) for each sample. O-PDX, orthotopic patient-derived xenograft. Tested protocols for processing each tumor type are indicated. **f**, Inferred CNA profiles for matched pre- and post-treatment neuroblastoma samples. Chromosomal amplification (red) and deletion (blue) inferred in each chromosomal position (columns) across the single cells (rows) from pre-treatment biopsy HTAPP-312-SMP-901 (left) and post-treatment resection HTAPP-312-SMP-902 (right). Top: reference cells not expected to contain CNAs in this tumor. Bottom: cells tested for CNAs relative to the reference cells. Color bars: assigned cell type signature for each cell. *n* = 1 sample per protocol. The numbers of cells (*k*) are indicated in **e**. Prot., protocol; NB, neuroblastoma; OPC, oligodendrocyte progenitor like cell; NPC, neural progenitor like cell; NK, natural killer; k., kit.

**Extended Data Fig. 1:**
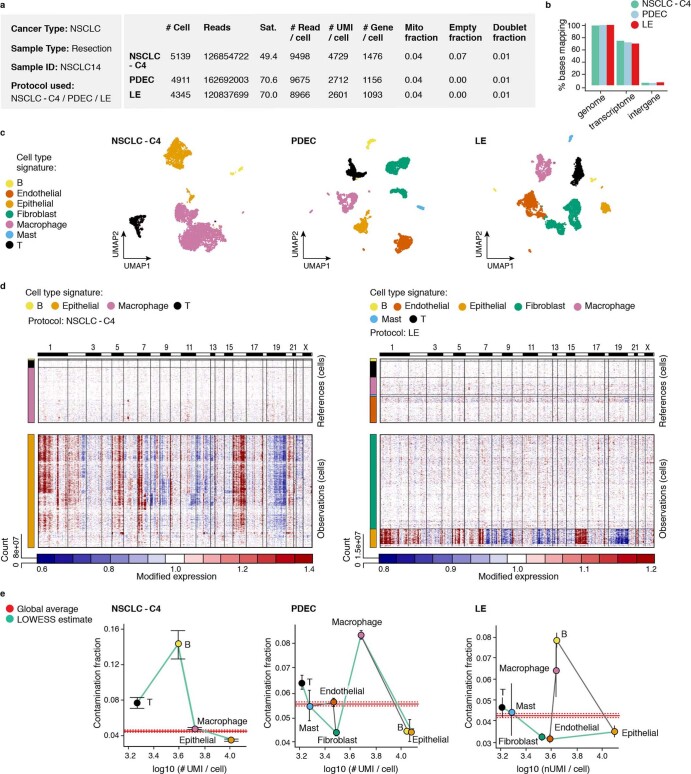
scRNA-Seq protocol comparison for a single NSCLC sample. **(a)** Sample processing and QC overview. For each protocol, shown are the number of cells passing QC, and the number of sequencing reads and sequencing saturation across all cells. The remaining metrics are reported for cells passing QC: the median number of reads per cell, median number of UMIs per cell, median number of genes per cell, median fraction of UMIs mapping to mitochondrial genes, fraction of cell barcodes called as empty droplets and fraction of cell barcodes called as doublets. **(b)** Read mapping QCs. The percent of bases in the sequencing reads (*y* axis) mapping to the genome, transcriptome and intergenic regions (*x* axis) across the three protocols (colored bars). **(c)** Cell type assignment. UMAP embedding of single cell profiles from each protocol colored by assigned cell type signature. **(d)** Inferred CNA profiles. Chromosomal amplification (red) and deletion (blue) inferred in each chromosomal position (columns) across the single cells (rows) from the NSCLC-C4 (left) and LE (right) protocols. Top: reference cells not expected to contain CNA in this cancer type. Bottom: cells tested for CNA relative to the reference cells. Color bar: assigned cell type signature for each cell. **(e)** Ambient RNA estimates. Estimates^18^ of the fraction of RNA in each cell type derived from ambient RNA contamination (y axis), with cell types ordered by their mean number of UMIs/cell (x axis). Red line: global average of contamination fraction; Green line: LOWESS (locally weighted scatterplot smoothing) smoothed estimate of the contamination fraction within each cell type, along with the associated binomial 95% confidence interval (Clopper–Pearson interval). *n* = 1 sample per protocol and number of cells (*k*) is indicated in (a).

**Extended Data Fig. 2:**
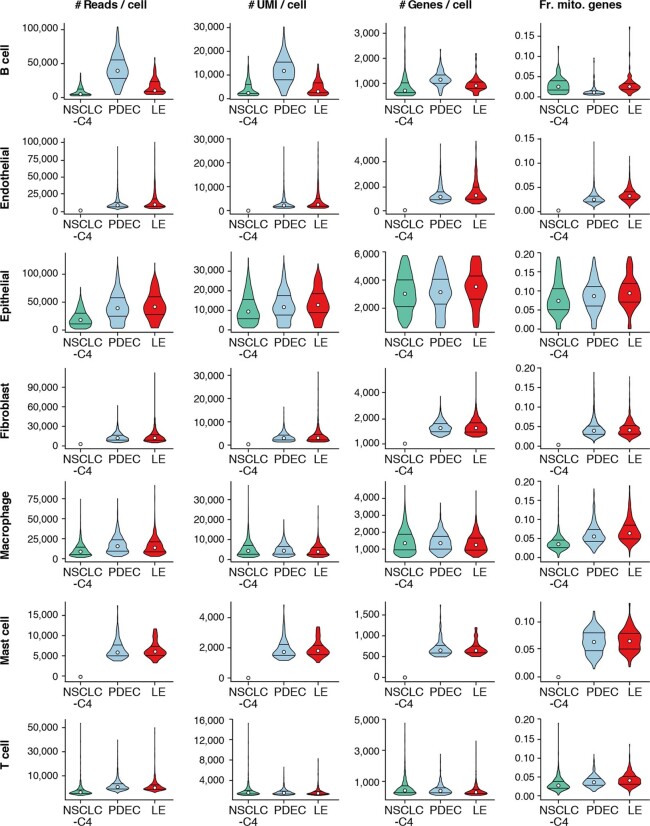
Cell type specific QC metrics for scRNA-Seq protocol comparison in a single NSCLC sample. Cell type specific QCs for NSCLC14. Distribution (median and first and third quartiles) of the number of reads per cell, number of UMIs per cell, number of genes per cell and fraction of UMIs mapping to mitochondrial genes in each cell (*y* axes) in each of the three protocols (*x* axis), for cells passing QC from each cell type (rows). *n* = 1 sample per protocol. Number of B cells (*k*) from NSCLC-C4, PDEC and LE, respectively, is: 100, 121, 78; endothelial cells: 0, 920, 1,078; epithelial cells: 1,284, 641, 260; fibroblasts: 0, 1,476, 1,403; macrophages: 3,306, 911, 727; mast cells: 0, 119, 77; T cells: 449, 723, 722.

**Extended Data Fig. 3:**
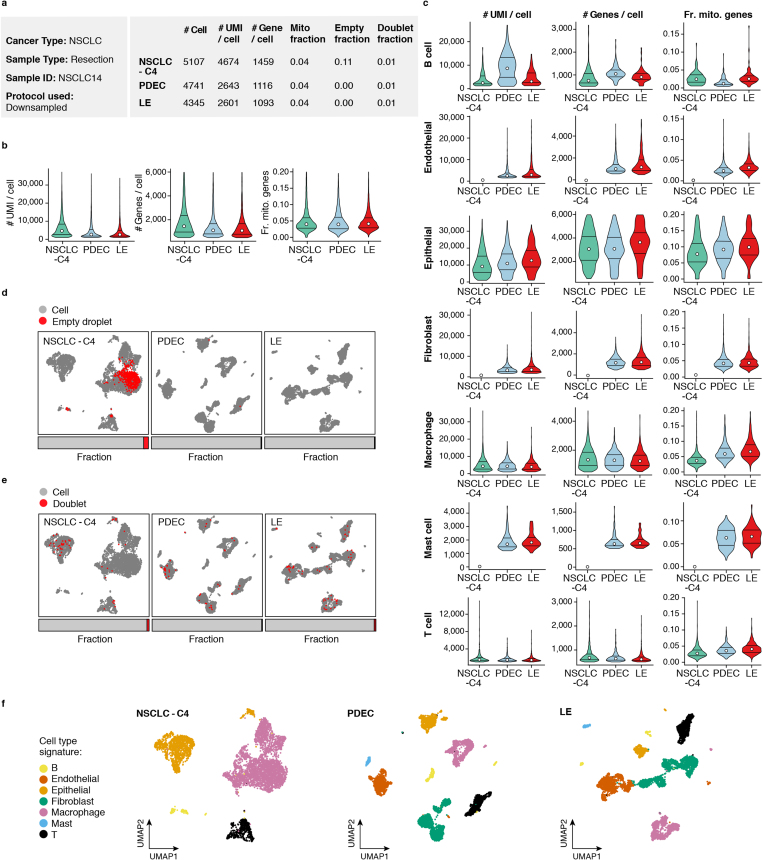
scRNA-Seq protocol comparison for NSCLC following read down-sampling. Shown are analyses for NSCLC14 (as in Extended Data Figs. 1 and 2), but after the total number of sequencing reads within each sample was down-sampled to match the protocol with the fewest total sequencing reads. **(a)** Sample processing and QC overview. For each protocol, shown are the number of cells passing QC. The remaining metrics are reported for those cells passing QC: median number of UMIs per cell, median number of genes per cell, median fraction of UMIs mapping to mitochondrial genes in each cell, fraction of cell barcodes called as empty droplets and fraction of cell barcodes called as doublets. **(b, c)** Overall and cell types specific QCs. Distribution (median and first and third quartiles) of the number of UMIs per cell, number of genes per cell and fraction of gene expression per cell from mitochondrial genes (*y* axes) in each of the three protocols (*x* axis), for all cells passing QC (b) and for cells from each cell type (c, rows). **(d,e)** Relation of empty droplets and doublets to cell types. UMAP embedding and fraction (horizontal bar) of single cell (gray), empty droplet (red, d) and doublet (red, e) profiles for each protocol **(f)** Cell type assignment. UMAP embedding of single cell profiles from each protocol colored by assigned cell type signature. *n* = 1 sample per protocol and number of cells (*k*) is indicated in (a). Number of B cells (*k*) from NSCLC-C4, PDEC and LE, respectively, is: 114, 157, 78; endothelial cells: 0, 879, 1,078; epithelial cells: 1,283, 644, 260; fibroblasts: 0, 1,439, 1,403; macrophages: 3,278, 853, 727; mast cells: 0, 106, 77; T cells: 432, 663, 722.

**Extended Data Fig. 4:**
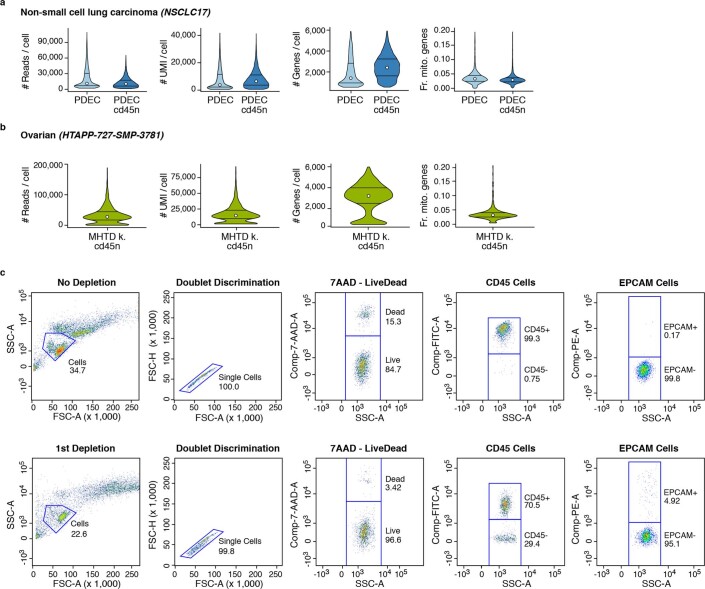
CD45^+^ depletion protocol enriches for non-immune cells in freshly processed NSCLC and ovarian ascites. **(a, b)** QCs. Distribution (median and first and third quartiles) of the number of reads per cell, number of UMIs per cell, number of genes per cell and fraction of gene expression per cell from mitochondrial genes (y axes) for all cells passing QC from NSCLC (a) before and after CD45^+^ cell depletion, and for ovarian ascites (*k* = 2,998 and 10,716 cells, respectively) or (b) after CD45^+^ cell depletion (2,359 cells) (x axis). *n* = 1 sample per protocol. **(c)** CD45^+^ cell depletion estimates in ovarian cancer ascites by FACS. Flow-cytometry comparison of single cells isolated without (top) or with (bottom) depletion of CD45^+^ cells. Cells were gated by FSC and SSC (first column), doublets removed using FSC-A and FSC-H (second column), 7-AAD gating of dead cells to identify live cells (third column), the distribution of immune and non-immune cells quantified using a CD45 antibody (fourth column) and the distribution of EPCAM^+^ cells quantified using an EPCAM antibody (fifth column). (Efficient removal of CD45^+^ cells from ovarian cancer ascites was also demonstrated with an independent sample from a different patient (data not shown).) Number of cells without and with depletion, respectively are: 10,000, 10,000 (1^st^ column), 3,468, 2,256 (2^nd^ column), 3,467, 2,251 (3^rd^ column), 2,936, 2,174 (4^th^ and 5^th^ columns).

As an example of the optimization process, consider the in-depth analysis of an NSCLC resection sample (sample NSCLC14, Fig. 2b–f and Extended Data Figs. 1–3). We used three processing protocols: (1) collagenase 4 (NSCLC-C4), (2) a mixture of pronase, dispase, elastase and collagenases A and 4 (PDEC) and (3) Liberase TM and elastase (LE), each in combination with DNase I (see Methods). For other tumor types, we show the results of selected protocols out of those tested (Figs. 1b, 2g and 3a–e and Extended Data Fig. 4a,b).

Protocols often performed similarly on standard QC measures (for example, number of cells recovered), but differed markedly in recovered cellular diversity or in the fraction of droplets predicted to contain only ambient RNA (‘empty drops’)—two evaluation criteria that we prioritized. For example, in the NSCLC14 resection sample, all methods yielded a similar number of cells with high-quality expression profiles (Fig. 2b,e and Extended Data Fig. 1a–c), doublets (Fig. 2c) and CNA patterns in malignant cells (Fig. 2f and Extended Data Fig. 1d). However, only the PDEC and LE protocols recovered fibroblasts and endothelial cells (Fig. 2e and Extended Data Fig. 1c), and NSCLC-C4 had a 100-fold higher fraction of droplets called as ‘empty’ (7% versus 0.08% and 0.04% in PDEC and LE, respectively; Fig. 2d and Extended Data Fig. 1a). The drops designated ‘empty’ in NSCLC-C4 clustered within macrophages (Fig. 2d and Extended Data Fig. 1c), the most prevalent cell type, suggesting that these cell barcodes either had lower sequencing saturation or that the sample itself had higher ambient RNA content. Although we estimated similarly low levels of ambient RNA^18^ across the three protocols (Extended Data Fig. 1e), NSCLC-C4 indeed had lower overall sequencing saturation (Extended Data Fig. 1a).

Comparing QC metrics across protocols can be challenging due to differences in cell type recovery and in sequencing depth between preparations, which we controlled for in the NSCLC14 sample by also evaluating QC metrics within each cell type and downsampling by total reads across protocols (Fig. 2b and Extended Data Fig. 2). For example, when we consider all cells in the NSCLC14 samples, NSCLC-C4 had a significantly higher number of detected genes (*P* = 1.3 × 10^−90^ versus PDEC; 1.4 × 10^−62^ versus LE, two-sided Mann–Whitney U test), but within B cells, PDEC had a significantly higher number of detected genes (*P* = 2 × 10^−15^ versus NSCLC-C4; 2 × 10^−10^ versus LE), whereas within epithelial cells, LE had the highest number (*P* = 5 × 10^−6^ versus NSCLC-C4; 2 × 10^−4^ versus PDEC) (Fig. 2b). Because the number of detected genes (and other metrics) varies between cell types, and cell type composition varies between the protocols (Fig. 2e), it is important to assess cell-type-specific QCs when selecting a protocol. Moreover, the lower sequencing saturation does not directly reflect the performance of the NSCLC-C4 protocol, and downsampling by total reads did not qualitatively change any of our protocol evaluation metrics (Extended Data Fig. 3). Considering all of these features, we selected the PDEC protocol for processing NSCLC tumor samples, as it balances cell type diversity and QCs per cell type.

### Fast depletion of immune cells for enrichment of malignant and stromal cells

Because in some tumor specimens the proportion of malignant cells is relatively low and that of immune cells is particularly high, we considered strategies to deplete CD45^+^ immune cells as a way to both enrich for epithelial cells without specific markers and to maintain any stromal cells. We chose to use MACS MicroBeads with anti-CD45 antibodies rather than sorting by flow cytometry (FACS), because samples are not always available at the designated time for which sorters are booked, and FACS requires longer sample processing, which may introduce additional cell stress, as we have found for epithelial cells (data not shown).

We optimized a CD45^+^ cell depletion strategy by testing different commercial kits and assessing the impact of one versus two rounds of depletion (data not shown). For example, we profiled an NSCLC tumor sample (NSCLC17) by scRNA-Seq before and after depletion, finding an increase from 26% to 82% epithelial cells (Fig. 2g and Extended Data Fig. 4a) and virtually no immune cells profiled post depletion. Similarly, in an ovarian ascites sample (HTAPP-727), we recovered 32% epithelial cells by scRNA-Seq post depletion (Fig. 2g and Extended Data Fig. 4b), compared to <1% CD45^−^EpCAM^+^ cells typically found in ovarian ascites by FACS (data not shown). Consistently, FACS shows that CD45^+^ cell depletion of another ascites sample increased the overall proportion of non-immune (CD45^−^) cells from 0.75% to 29.4% and increased the proportion of EpCAM^+^ cells from 0.17% to 4.9% (Extended Data Fig. 4c).

### Successful scRNA-Seq of biopsies and post-treatment samples from diverse tumors

We successfully applied the scRNA-Seq toolbox to much smaller core biopsy clinical samples from different anatomical sites. For example, in MBC, we applied the LD (Liberase TM and DNase I) protocol to a resection (HTAPP-254) and a biopsy (HTAPP-735) from lymph node metastases from two patients, yielding similarly successful QCs (Fig. 3a–d). The resection and biopsy of the two patients had different cellular compositions (Fig. 3e): the biopsy had a higher proportion of epithelial, endothelial and fibroblast cells and a lower proportion of T cells compared to the resection. We similarly successfully profiled biopsies of MBC liver metastases (HTAPP-285 and HTAPP-963) with the same protocol (Fig. 3a–d), recovering some hepatocytes in addition to a similar range of cell types as was recovered in the lymph node biopsy (Fig. 3e). Thus, this protocol can be used across breast cancer metastases from different anatomical metastatic sites.

The scRNA-Seq toolbox also performs well on samples obtained post-treatment, which can pose challenges as a result of cell death and changes in cell type composition with treatment. For example, both a pre-treatment diagnostic biopsy (HTAPP-312-pre) and a post-treatment resection (HTAPP-312-post) from the same neuroblastoma patient profiled with the NB-C4 protocol yielded high QCs (Fig. 3a–d) and similar CNA patterns in malignant cells (Fig. 3f). More cells, but of fewer cell types, were recovered in the pre-treatment biopsy (4,369 cells: neuroendocrine, T cells and macrophages) than the post-treatment resection (786 cells: neuroendocrine, T cells, macrophages, as well as endothelial cells and fibroblasts) (Fig. 3e), consistent with observed post-treatment fibrosis. We tested an additional dissociation protocol (papain) in a neuroblastoma O-PDX sample (O-PDX1)^19,20^, which is not expected to include non-malignant human cells and indeed resulted in high-quality malignant cell profiles (Fig. 3a–e). The experimental QCs for the papain protocol were superior to those we had observed in other samples with NB-C4, a trend we corroborated in additional neuroblastoma samples (data not shown). Thus, we ultimately selected the papain protocol for neuroblastoma tumors.

In addition to such NSCLC, ovarian cancer ascite, MBC and neuroblastoma samples, we established effective scRNA-Seq protocols for GBM (GBM125), CLL (CLL1) and ovarian cancer tumors (HTAPP-624) (Fig. 3a–e). In particular, in CLL, we successfully recovered the expected cell types from a cryopreserved sample, containing viable cells. This reflects the increased resilience of immune cells to freezing compared to other cell types, also observed in other settings^21^, and the lack of a dissociation step in CLL scRNA-Seq (see Methods). Cryopreservation, however, can increase the proportion of damaged cells^22^ and may not successfully recover all the malignant and other non-malignant cells in the tumor.

### Four nucleus isolation protocols assessed for snRNA-Seq of frozen tumors

**Fig. 4:**
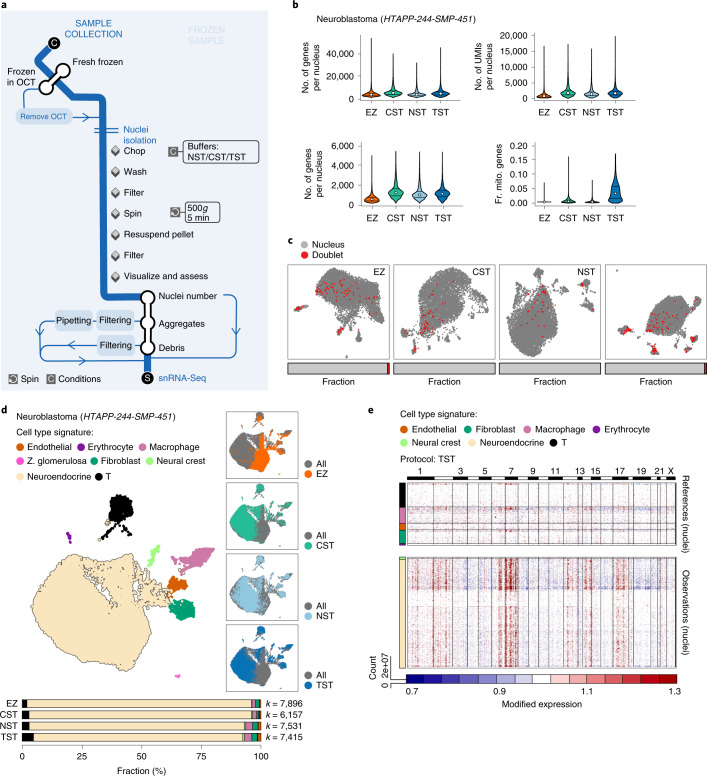
Frozen tumor processing and protocol selection for snRNA-Seq. **a**, Flow chart for collection and processing of frozen tumor samples. **b**–**e**, Comparison of four nucleus isolation protocols in one neuroblastoma sample. **b**, Variation in protocol performance: distributions (median and first and third quartiles) of the number of UMIs per nucleus, the number of genes per nucleus and fraction of UMIs per nucleus mapping to mitochondrial genes (*y* axes) in each protocol (*x* axis) across all nuclei in the dataset. **c**, The protocols detect similar numbers of doublets. UMAP embedding of single nucleus profiles (dots) for each protocol is colored by assignment as nucleus (gray) or doublet (red). Horizontal bars (bottom): fraction of single (gray) and doublet (red) nuclei. **d**, The protocols vary in the diversity of cell types captured. UMAP embedding of single nucleus profiles (dots) from all four protocols is colored by assigned cell subset signature (left) or protocol (right). Bottom: proportion of cells from each subset in each of the protocols. *k*, number of nuclei passing QC. **e**, Inferred CNA profiles. Chromosomal amplification (red) and deletion (blue) inferred in each chromosomal position (columns) across the single nuclei (rows) from the TST protocol are shown. Top: reference nuclei not expected to contain CNAs in this tumor. Bottom: nuclei tested for CNAs relative to the reference nuclei. Color bar: assigned cell type signature for each nucleus. *n* = 1 sample per protocol. The numbers of nuclei (*k*) are indicated in **d**. OCT, optimal cutting temperature compound.

For frozen specimens from solid tumors, we optimized snRNA-Seq, assessing different methods for nucleus isolation (Fig. 4a and Methods) across seven tumor types: neuroblastoma, MBC, ovarian cancer, pediatric sarcoma, melanoma, pediatric high-grade glioma and CLL (Fig. 1b). We initially apportioned larger samples or used multiple biopsies to compare four isolation methods: EZPrep^8^, Nonidet P40 with salts and Tris (NST) (modified from ref. ^23^), CHAPS, with salts and Tris (CST)^11^, and Tween with salts and Tris (TST)^11^. The methods differ primarily in the mechanical force (for example, chopping or douncing), buffer (EZ versus ST) and/or detergent composition (see Methods). Because in early tests EZPrep routinely underperformed CST, NST and TST (data not shown), we only included EZPrep in initial comparisons (below). We used single nucleus suspensions from all tested protocols as input for droplet-based snRNA-Seq (see Methods), thus sampling sufficiently large numbers of cells to evaluate cell diversity and QCs.

To evaluate protocols, we used the post hoc computational criteria above (Fig. 1a), except we excluded the estimation of empty drops because it was only developed and tested on single-cell RNA-Seq data. We further customized Cumulus^14^ for snRNA-Seq data, mapping reads to both exons and introns, and adapted the QC thresholds for transcript (UMI) and gene counts to reflect the lower expected mRNA content in nuclei (see Methods). Experimentally, we added in-process light microscopy QCs to ensure complete nucleus isolation and to estimate doublets, aggregates and debris (Fig. 4a and Methods). As with scRNA-Seq, we tested protocols several times to confirm similar performance trends.

### TST protocol typically recovers the highest diversity of cell types

**Fig. 5:**
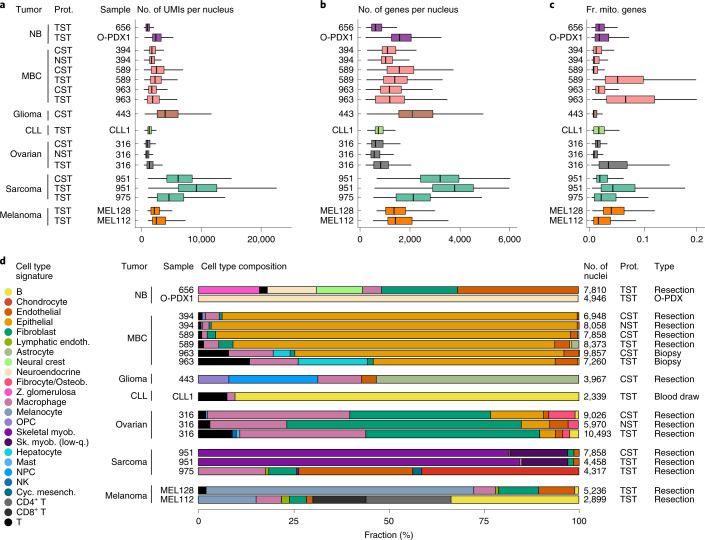
snRNA-Seq protocol comparison across tumor types. **a**–**c**, QC metrics: distributions (median and first and third quartiles) of the number of UMIs per nucleus (**a**), the number of genes per nucleus (**b**) and the fraction of UMIs per nucleus mapping to mitochondrial genes (**c**) (*x* axes) for each sample (*y* axis). **d**, Cell type composition, showing the proportion of nuclei assigned to each cell type signature (color) for each sample. *n* = 1 sample per protocol. The numbers of nuclei (*k*) are indicated in **d**.

**Extended Data Fig. 5:**
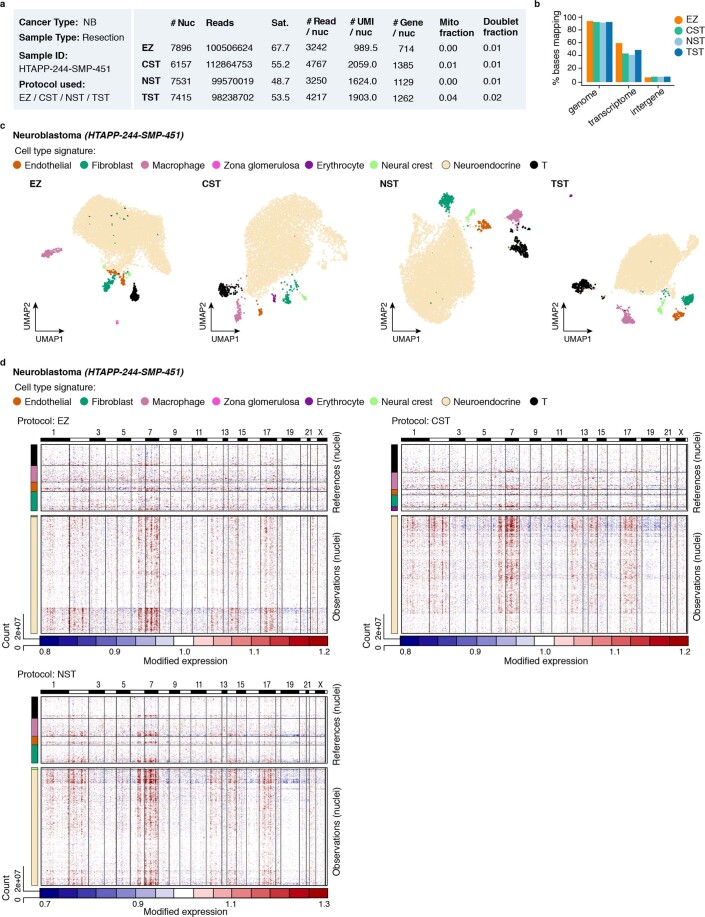
snRNA-Seq protocol comparison in a single neuroblastoma sample. (**a)** Sample processing and QC overview. For each protocol, shown are the number of nuclei passing QC, number of sequencing reads and sequencing saturation across all nuclei. The remaining metrics are reported for those nuclei passing QC: median number of reads per nucleus, median number of UMIs per nucleus, median number of genes per nucleus, median fraction of UMIs mapping to mitochondrial genes in each nucleus and fraction of nucleus barcodes called as doublets. **(b)** Read mapping QCs. The percent of bases in the sequencing reads (*y* axis) mapping to the genome, transcriptome and intergenic regions (*x* axis) across the four protocols (colored bars). **(c)** Cell type assignment. UMAP embedding of single nucleus profiles from each protocol colored by assigned cell type signature. **(d)** Inferred CNA profiles. Chromosomal amplification (red) and deletion (blue) inferred in each chromosomal position (columns) across the single nuclei (rows). Top: reference nuclei not expected to contain CNA in this cancer type. Bottom: nuclei tested for CNA relative to the reference nuclei. Color bar: assigned cell type signature for each nucleus. *n* = 1 sample per protocol and number of nuclei (*k*) is indicated in (a).

**Extended Data Fig. 6:**
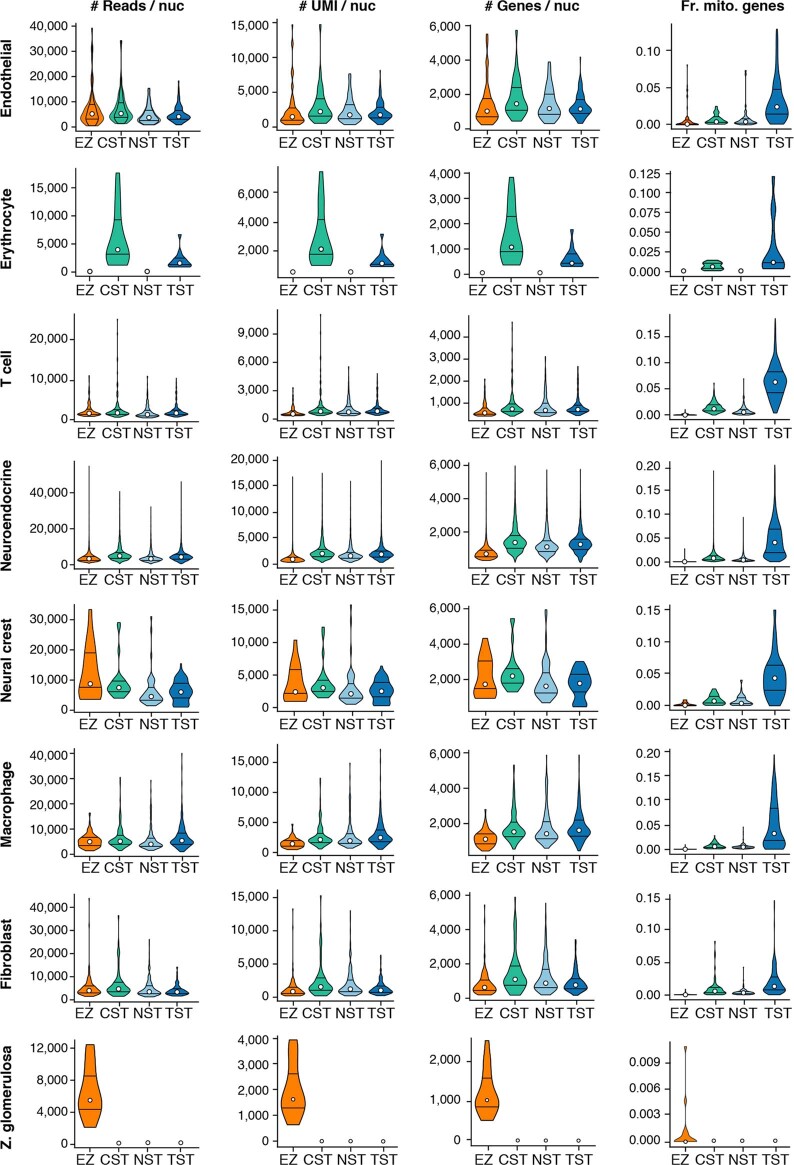
Cell type specific QC metrics for snRNA-Seq protocol comparison in a single neuroblastoma sample. Cell type specific QCs for HTAPP-244-SMP-451. Distribution (median and first and third quartiles) of the number of reads per nucleus, number of UMIs per nucleus, number of genes per nucleus and fraction of UMIs mapping to mitochondrial genes in each nucleus (*y* axes) in each of the four protocols (*x* axis), for nuclei passing QC from each cell type (rows). *n* = 1 sample per protocol. Number of endothelial nuclei (*k*) from EZ, CST, NST and TST, respectively, is: 69, 32, 91, 95; erythrocyte: 0, 18, 0, 15; T cell: 157, 171, 229, 337; neuroendocrine: 7,379, 5,728, 6,790, 6,477; neural crest: 18, 27, 50, 67; macrophage: 119, 107, 189, 230; fibroblast: 138, 74, 182, 194; zona glomerulosa: 16, 0, 0, 0.

**Extended Data Fig. 7:**
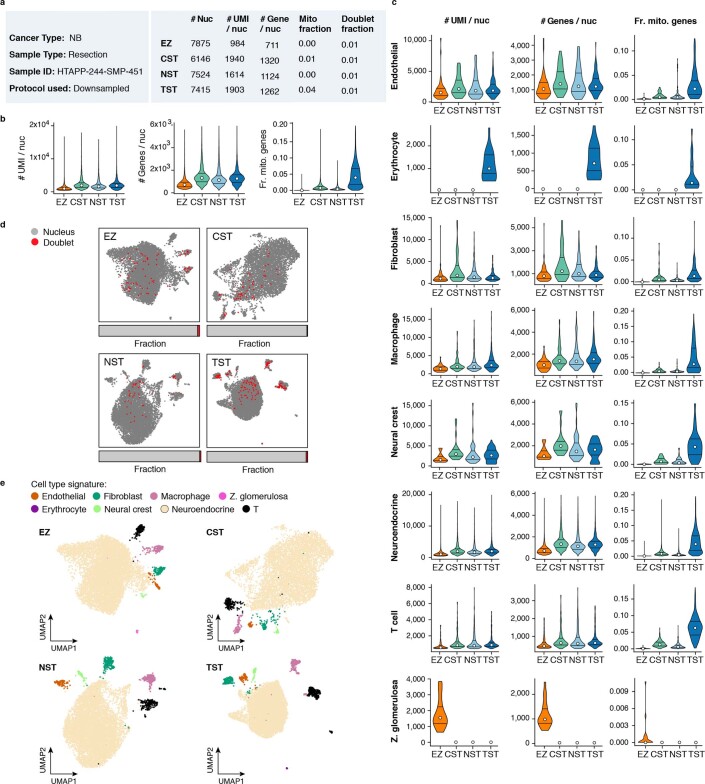
snRNA-Seq protocol comparison in a single neuroblastoma sample following read down-sampling. Shown are analyses for NB HTAPP-244-SMP-451 (as in Extended Data Figs. 5 and 6), but after the total number of sequencing reads within each sample was down-sampled to match the protocol with the fewest total sequencing reads. **(a)** Sample processing and QC overview. For each protocol, shown are the number of nuclei passing QC. The remaining metrics are reported for those nuclei passing QC: median number of UMIs per nucleus, median number of genes per nucleus, median fraction of UMIs mapping to mitochondrial genes in each nucleus and fraction of nucleus barcodes called as doublets. **(b,c)** Overall and cell types specific QCs. Distribution (median and first and third quartiles) of the number of UMIs per nucleus, number of genes per nucleus and fraction of UMIs mapping to mitochondrial genes in each nucleus (*y* axes) in each of the four protocols (*x* axis), for all nuclei passing QC (b) and for nuclei from each cell type (**c**, rows). **(d)** Relation of doublets to cell types. UMAP embedding and fraction (horizontal bar) of single nucleus (gray) and doublet (red) profiles for each protocol. **(e)** Cell type assignment. UMAP embedding of single nucleus profiles from each protocol colored by assigned cell type signature. *n* = 1 sample per protocol and number of nuclei (*k*) is indicated in (a). Number of endothelial nuclei (*k*) from EZ, CST, NST, TST is: 53, 31, 95, 98; erythrocyte: 0, 0, 0, 23; T cell: 146, 177, 230, 345; neuroendocrine: 7,407, 5,726, 6,776, 6,454; neural crest: 14, 27, 50, 69; macrophage: 123, 104, 196, 240; fibroblast: 111, 81, 177, 186; zona glomerulosa: 21, 0, 0, 0.

Overall, three nucleus isolation methods—TST, CST and NST—had comparable performances based on the assessed nucleus quality (Figs. 4b–d and 5 and Extended Data Figs. 5 and 6), with TST typically yielding the greatest cell type diversity and number of nuclei per cell type, together with the highest expression of mitochondrial genes, and NST typically having the fewest genes per nucleus and lowest diversity of cell types. For example, in neuroblastoma, testing each of the four protocols on a single resection sample (HTAPP-244) yielded a similar number of high-quality nuclei (7,896, 6,157, 7,531 and 7,415 for EZ, CST, NST and TST, respectively) (Fig. 4b–d and Extended Data Fig. 5a–c), nucleus doublets (Fig. 4c), cell types—with malignant neuroendocrine cells being the most prevalent (Fig. 4d and Extended Data Fig. 5c) and malignant cells with similarly detectable CNAs (Fig. 4e and Extended Data Fig. 5d; CNAs are less prominent, as expected for this pediatric, low-risk sample). Nuclei prepared with the EZ protocol had lower numbers of UMIs and genes detected (Fig. 4b) compared to the three ST protocols. TST recovered more endothelial cells, fibroblasts, neural crest cells and T cells than the other protocols (Fig. 4d). TST also yielded a higher expression of mitochondrial genes (Fig. 4b), in this and all other tumors tested (Fig. 5c), because the nuclear membrane, endoplasmic reticulum and ribosomes remain attached to the nucleus when using this method^11^. The same trends were preserved in cell-type-specific QCs (Extended Data Fig. 6) and after downsampling by the total number of sequencing reads (Extended Data Fig. 7).

**Extended Data Fig. 8:**
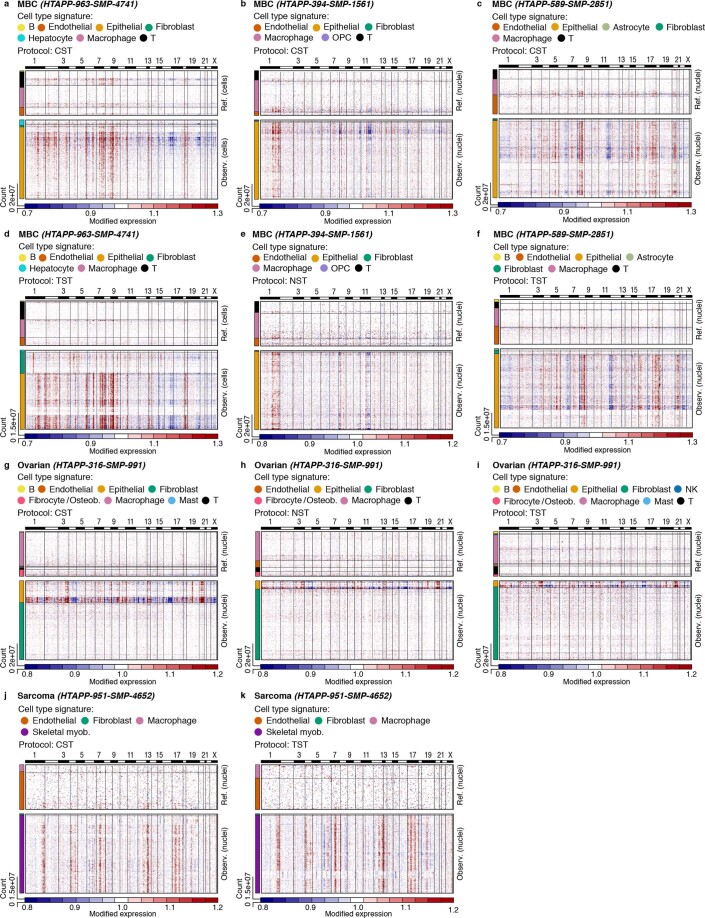
Inferred CNA profiles from snRNA-Seq in diverse tumors. Chromosomal amplification (red) and deletion (blue) inferred in each chromosomal position (columns) across the single nuclei (rows) from three MBC samples (**a–f**), one ovarian cancer sample (**g-i**) and one sarcoma sample (**j, k**). Top: reference nuclei not expected to contain CNA in this cancer type. Bottom: nuclei tested for CNA relative to the reference nuclei. Color bar: assigned cell type signature for each nucleus. *n* = 1 sample per protocol and number of nuclei (*k*) per sample: MBC HTAPP-963-SMP-4741—9,857 (CST), 7,260 (TST); MBC HTAPP-394-SMP-1561—6,948 (CST), 8,058 (NST); MBC HTAPP-589-SMP-2851—7,858 (CST), 8,373 (TST); ovarian HTAPP-316-SMP-991—9,026 (CST), 5,970 (NST), 10,493 (TST); sarcoma HTAPP-951-SMP-4652—7,858 (CST), 4,458 (TST).

The CST, NST and TST nucleus isolation methods had similar performance characteristics when tested with MBC, ovarian cancer and pediatric sarcoma samples, with TST providing the most diversity in cell types, especially in non-malignant cells. In MBC, we compared CST and NST in one metastatic brain resection (HTAPP-394) and CST and TST in another metastatic brain resection (HTAPP-589) and in a metastatic liver biopsy (HTAPP-963) (Fig. 5). In all cases, QC statistics (Fig. 5a–c) and CNA patterns (Extended Data Fig. 8a–f) were similar between protocols, and nuclei from epithelial cells were the most prevalent (Fig. 5d). CST and NST captured a very similar distribution of cell types, while TST captured more non-malignant cells, including T cells (Fig. 5d), and a higher fraction of mitochondrial reads (Fig. 5c). In ovarian cancer, CST, NST and TST recovered similar CNA patterns from the same sample (HTAPP-316; Extended Data Fig. 8g–i), but TST captured the greatest cell type diversity (Fig. 5d), whereas NST recovered fewer nuclei, genes per nucleus and UMIs per nucleus (Fig. 5a–c), and had a lower cell type diversity (Fig. 5d), despite having greater overall sequencing depth (73% sequencing saturation versus 57% in CST and 50% in TST). In a rhabdomyosarcoma sample (HTAPP-951), CST and TST captured the same cell types at similar proportions (Fig. 5d) and showed similar CNA patterns (Extended Data Fig. 8j,k).

**Extended Data Fig. 9:**
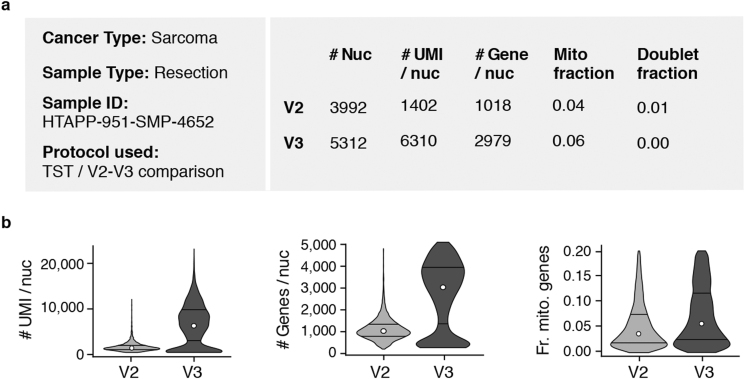
snRNA-Seq protocol comparison of V2 and V3 chemistry from 10x Genomics on a resection of sarcoma. **(a)** Sample processing and QC overview. For each protocol, shown are the number of nuclei passing QC, after the total number of sequencing reads from the V3 protocol data was down-sampled to match the number of reads in the V2 data. The remaining metrics are reported for those nuclei passing QC: median number of UMIs per nucleus, median number of genes per nucleus, median fraction of UMIs mapping to mitochondrial genes in each nucleus and fraction of nucleus barcodes called as doublets. **(b)** Overall QCs. Distribution (median and first and third quartiles) of number of UMIs per nucleus, number of genes per nucleus and fraction of UMIs mapping to mitochondrial genes in each nucleus (y axes) for all nuclei passing QC. *n* = 1 sample per chemistry type and number of nuclei (*k*) is indicated in (a).

Overall, we chose the TST protocol for most tumor types and CST for tumors from neuronal tissues, such as pediatric high-grade glioma. With the protocols we selected (Fig. 1b, right column), we profiled additional neuroblastoma tumors (HTAPP-656, O-PDX1) as well as Ewing sarcoma (HTAPP-975), melanoma (MEL112, MEL128), pediatric high-grade glioma (HTAPP-443) and CLL (CLL1) tumor samples—spanning biopsies, resections and treated samples (Figs. 1b and 5). We also tested a pediatric rhabdomyosarcoma sample (HTAPP-951) by two different chemistries for droplet-based snRNA-Seq (V2 versus V3 from 10x Genomics; see Methods), obtaining, overall, similar results in terms of cell types detected, but an improved number of recovered versus expected nuclei and higher complexity per nucleus in V3 (Extended Data Fig. 9).

### Different cell composition recovered by scRNA-Seq and snRNA-Seq

**Fig. 6:**
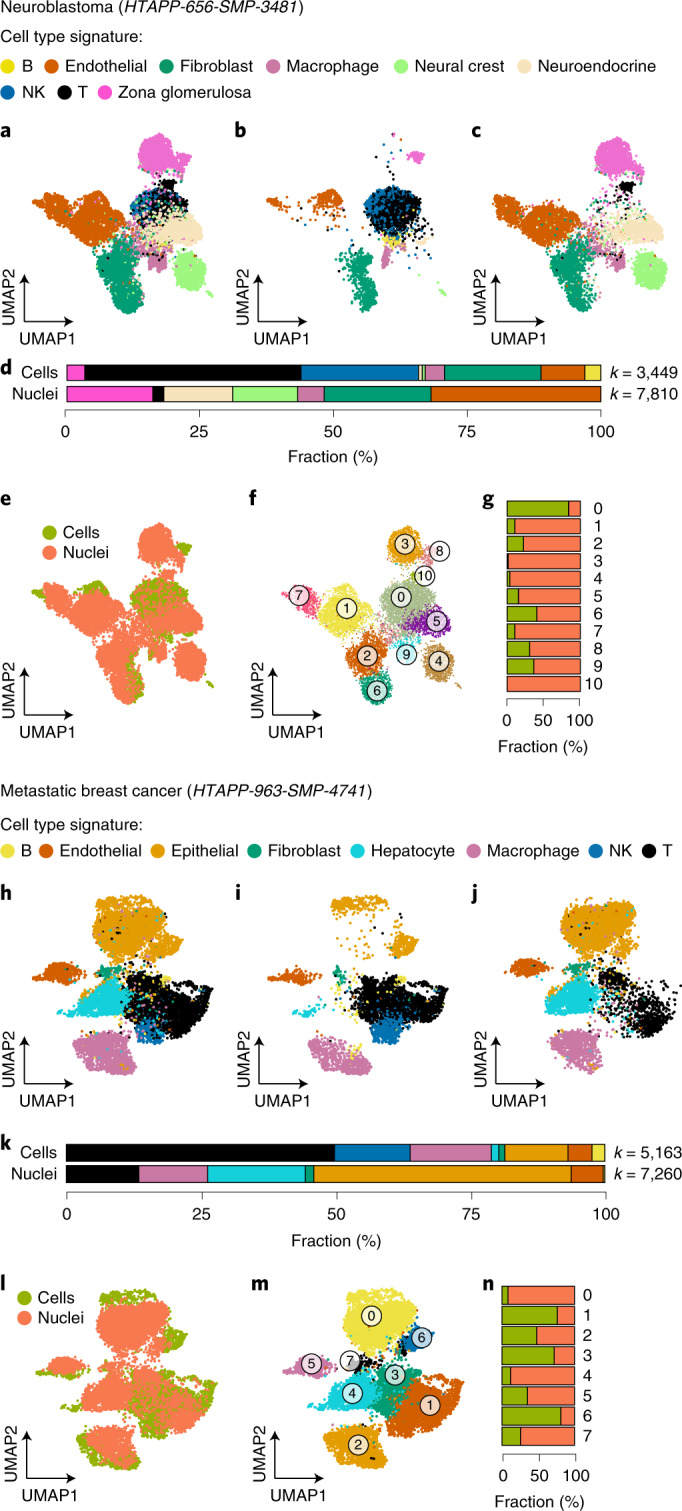
scRNA-Seq and snRNA-Seq recover comparable cells in different proportions. **a**–**g**, Neuroblastoma. **a**–**c**, UMAP embedding of scRNA-Seq and snRNA-Seq profiles of the same neuroblastoma sample combined by CCA^24^ (Methods) showing profiles (dots) from both (**a**), scRNA-Seq (**b**) and snRNA-Seq (**c**), colored by the assigned cell type signatures. **d**, Proportion of cells from each subset in the two protocols. *k*, number of cells or nuclei passing QC. **e**,**f**, Same UMAP embedding as in **a**, colored by cells or nuclei (**e**) or by unsupervised clustering (**f**). **g**, Fraction of cells and nuclei in each cluster from **f**. *n* = 1 sample per protocol. The numbers of cells and nuclei (*k*) are indicated in **d**. **h**–**n**, MBC. As in **a**–**g** for an MBC sample. *n* = 1 sample per protocol. The numbers of cells and nuclei are indicated in **k**.

**Extended Data Fig. 10:**
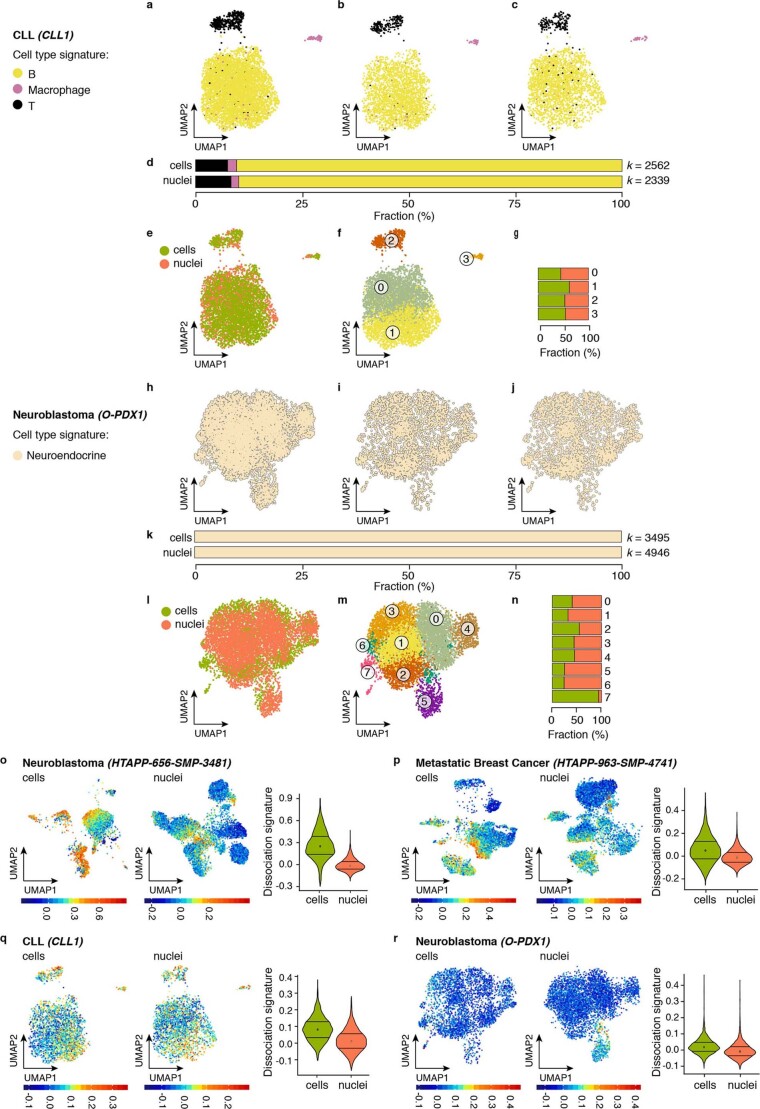
Comparison of scRNA-Seq and snRNA-Seq from the same tumor sample. (**a–g**) CLL. UMAP embedding of scRNA-Seq and snRNA-Seq profiles of the same CLL sample combined by CCA^24^ (**Methods**) showing profiles (dots) from both (a), scRNA-Seq (b) and snRNA-Seq (c), colored by assigned cell type signatures. (d) Proportion of cells from each subset in the two protocols. *k*: number of cells or nuclei passing QC. (e-f) Same UMAP embedding as in (a), colored by cells or nuclei (e) or unsupervised clustering (f). (g) Fraction of cells and nuclei in each cluster. *n* = 1 sample per protocol and number of cells and nuclei is indicated in (d). (**h–n**) O-PDX neuroblastoma. As in (a-g) for an O-PDX neuroblastoma sample. *n* = 1 sample per protocol and number of cells and nuclei is indicated in (k). (**o-r**) Dissociation signatures are more prominent in cells than in nuclei from the same tumors. Left and middle: UMAP embedding of scRNA-Seq (left) and snRNA-Seq (middle) profiles (dots) of the same tumor combined by CCA^24^ and colored by the score of a dissociation signature (color bar). Right: Distribution of dissociation signature score (y axis; median and first and third quartiles) in cells (green) and nuclei (orange). (o) neuroblastoma (3,449 cells, 7,810 nuclei), (p) MBC (5,163 cells, 7,260 nuclei), (q) CLL (2,562 cells, 2,339 nuclei), (r) O-PDX neuroblastoma (3,495 cells, 4,946 nuclei).

We compared scRNA-Seq and snRNA-Seq by testing matching samples from the same specimen each in neuroblastoma (HTAPP-656, Fig. 6a–g), MBC (HTAPP-963, Fig. 6h–n), CLL (CLL1, Extended Data Fig. 10a–g) and O-PDX (O-PDX1, Extended Data Fig. 10h–n). The two approaches typically recovered similar cell types, but sometimes at varying proportions. In both neuroblastoma and MBC, immune cells were much more prevalent in scRNA-Seq, and parenchymal (especially malignant) cells were much more prevalent in snRNA-Seq (Fig. 6a–d,h–k). In all tested tumor types, cells and nuclei readily aligned following batch correction by canonical correlation analysis (CCA^24^, see Methods), grouping by cell type (Fig. 6e–g,l–n and Extended Data Fig. 10e–g,l–n).

Finally, we leveraged the matched data to examine the extent to which expression patterns in cells from dissociated fresh tissue indicate specific stress and compared these to those in nuclei from snap-frozen tissue. To this end, we scored each cell or nucleus in the matched data from recently published ‘dissociation signatures’^25^ (Extended Data Fig. 10o–r). In general, dissociation signatures were detectable in a larger proportion of cells than of nuclei, especially in solid tumors (neuroblastoma and MBC), and scored significantly higher in cells (*P* < 1 × 10^−100^, two-sided Mann–Whitney U test). When present in nuclei, however, these nuclei are embedded in the same regions of the phenotypic space as high scoring cells. Notably, in both cells and nuclei, the signature is more prominent in immune cells (for example, T, NK and macrophages) and stroma cells (for example, fibroblasts and endothelial). Although this may be a signature of damage from dissociation in some parenchymal cells, it is also probably a signature of immune activation and the immediate early response more generally. As a result, the interpretation of a ‘dissociation signature’ derived in a distinct setting must be done with extreme care, accounting for its cell (rather than nucleus) data source and its relation to immediate early gene expression (a native response in situ as well).

## Discussion

Single-cell genomics of clinical tumor specimens obtained as part of routine disease management or through research biopsies should help guide new discoveries and better deployment of therapies^26,27^, as initial studies have shown in the context of gliomas^6,17^, melanoma^3,28^, head and neck squamous cell carcinoma^4,29^ and other malignancies^30–34^. However, this requires adaptation of laboratory protocols, initially developed in research settings and requiring very rapid handling of fresh tissue, into the context of clinical sample acquisition and processing. Indeed, these initial studies were applied to small numbers of samples, often from resections, or focused on readily isolated immune cells. Analyzing the larger numbers of samples that would be required in the context of a clinical trial or longitudinal research, involving multiple sites, calls for streamlined and robust protocols. Moreover, systematically characterizing the diverse cells in solid tumors requires robust recovery of cells, many of which, including malignant epithelial cells, are highly sensitive. These challenges are further compounded by the diversity of tissues in which tumors and metastases reside.

Here, we take on these challenges by developing a systematic toolbox for protocols for single-cell and single-nucleus RNA-Seq, with detailed workflows and protocols from sample acquisition to library preparation for processing fresh and frozen clinical tumor samples across eight tumor types, as well as guidelines for testing and selecting protocols for processing future clinical tumor samples. We provide computational pipelines for extensive QCs at https://github.com/klarman-cell-observatory/HTAPP-Pipelines, and all laboratory protocols are provided in detailed form in the open access platform protocols.io.

When selecting a protocol for fresh tissue dissociation, we suggest testing two to three dissociation methods, chosen based on tumor type and tissue composition, and processing according to the fresh sample decision tree (Fig. 2a). We selected the best performing protocol by assessing both experimental and computational QC metrics, and, if desired, added a depletion step. When selecting a protocol for frozen tissues, we suggest testing the NST, TST and CST protocols, and processing according to our snRNA-Seq workflow (Fig. 4a). Although TST is often favorable due to its ability to capture the most diverse set of cells, in some tumors we recommend CST or NST (for example, CST for pediatric high-grade glioma; Fig. 1b). CST also yields fewer mitochondrial reads, reducing sequencing cost. For both sc- and snRNA-Seq, it is important to evaluate the selected protocol on multiple samples to ensure consistent performance, given the inherent variation in tumors.

Although we indicate the protocol we ultimately selected for the eight tumor types tested, the optimal protocol could be different for other studies due to sample characteristics and research questions. First, each patient and each sample are different, and researchers must strike a balance between a uniform protocol and realistic expectations of success. Second, the ‘ideal’ protocol depends on the research goals. As we show, most protocols vary in cell recovery, and it is not clear which, if any, provides the full ground truth of cellular composition. Moreover, even when one protocol does provide a faithful cellular composition, a researcher may opt for another approach. For example, some researchers may want to detect as many cell types as possible (and may favor one that enriches rare cells), others may be interested in a specific category of cells and opt for the one that is most successful in their recovery and yet others would want to compare cell proportions across tumors and would want their most faithful representation. Our decision trees will help researchers in making informed choices best suited to their samples and questions.

When researchers set out to test new protocols, several principles can help in experimental design. First, because clinical samples are often inherently limiting, benchmarking and technical development often cannot be performed on a single matched sample. This is especially the case for fresh sample dissociation, because it requires both larger input specimens and very rapid processing. In this case, we suggest testing different protocols across several samples. Researchers may also choose one of the protocols we presented as a starting point for further optimization. For fresh tissue, researchers should first evaluate tissue dissociation by in-process QCs, especially cell viability and extent of dissociation to single-cell suspension, and only samples that pass those should proceed to scRNA-Seq (for example, in MBC we have previously ruled out the use of Accumax for dissociation (data not shown)). FACS could also be used prior to profiling as an additional QC. It is easier to perform a side-by-side evaluation of nucleus isolation protocols, because they require smaller portions of tissue and can be started at a convenient time. However, in-process experimental QCs for nuclei are less informative and snRNA-Seq is typically required to assess performance.

Because scRNA-Seq and snRNA-Seq vary in their recovered cellular compositions, it is advantageous, when possible, to analyze both fresh and frozen tumor samples. The choice between scRNA-Seq and snRNA-Seq is typically driven by sample availability, logistics and biological questions. scRNA-Seq measures the expression in the whole cell, and the intact cell membrane allows for selection of specific cellular populations and protein profiling by CITE-Seq^35^. snRNA-Seq decouples sample procurement from processing, recovers nuclei from hard-to-dissociate samples (for example, bone, adipose and liver), and allows multiplexing of samples accrued over time^36,37^, including from banks. This can aid in sample selection and experimental design, reduce batch effects and open the study of rare or unusual samples that may be collected from many sites.

Our toolbox will help researchers systematically profile additional human tumors, leading to a deeper understanding of tumor biology. The toolbox will support charting of high-resolution tumor cell atlases^7^, which will yield insights that inform clinical work and should help improve precision in diagnostics and therapeutics.

## Methods

### Experimental methods

#### Human patient samples

External sample cohorts were added to the Broad Institute’s Molecular Classification of Cancer protocol (15–370B) and reviewed and approved by the Dana-Farber Cancer Institute’s (DFCI) Institutional Review Board (IRB). No subject recruitment or ascertainment was performed as part of the Broad protocol. Samples added to this protocol also underwent IRB review and approval at the institutions where the samples were originally collected. Specifically, DFCI IRB approved the following protocols: NSCLC (IRB protocol 98-063), MBC (IRB protocol 05-246), neuroblastoma (IRB protocols 11-104 and 17-104), ovarian cancer (IRB protocol 02-051), melanoma (IRB protocol 11-104), sarcoma (IRB protocol 17-104), GBM (IRB protocol 10-417) and CLL (IRB protocol 99-224), and the St Jude Children’s Research Hospital IRB approved the following protocol: pediatric high-grade glioma (IRB protocol 97BANK).

The XPD 09-234 MAST (Molecular Analysis of Solid Tumor) protocol for creating the neuroblastoma O-PDX sample was reviewed and approved by the St Jude Children’s Research Hospital IRB.

#### Laboratory animals

For the neuroblastoma O-PDX sample, animal use was restricted to one female nude athymic mouse for para-adrenal injection of O-PDX cells. This study was carried out in strict accordance with the recommendations in the Guide to Care and Use of Laboratory Animals of the National Institute of Health. The protocol was approved by the Institutional Animal Care and Use Committee at St Jude Children’s Research Hospital. All efforts were made to minimize suffering. All mice were housed in accordance with approved IACUC protocols. Animals were housed on a 12–12 h light cycle (light on 6:00 and off 18:00) and provided food and water ad libitum. Athymic nude female mice were purchased from Charles River Laboratories (strain code 553).

#### Collection of fresh tissue for scRNA-Seq

Collection of fresh solid tumor tissue for NSCLC, ovarian cancer and MBC at Brigham and Women’s Hospital (BWH)/DFCI was performed following protocols established to reduce the time elapsed between removal of the tumor tissue from the body, placement of the specimen in media and processing for scRNA-Seq. To this end, we established procedures between the hospital team (surgeon/clinical research coordinator/clinical pathologist), the coordinating team (project managers/pathology technician) and the processing team (staff scientists/research technicians) before procedure day. This included providing the hospital team with collection containers with appropriate media and predefining allocation priorities to ensure quick handling by the pathology technician of the sample received. On the day of the procedure, timely communication between the teams ensured quick specimen transfer from the hospital team to the research team, timely transport to the Broad Institute for processing, and immediate loading of the single cell suspension into the 10x Genomics Single-Cell Chromium Controller (as described in the section ‘Dissociation workflow from fresh solid tumor samples’ below).

In all cases, the tissue received from the hospital team was examined by the research pathology technician and, following procurement of a specimen for anatomic pathology review, the highest-quality portion (or core) was allocated for scRNA-Seq, placed in medium and transported to the Broad Institute for dissociation following the appropriate protocol (protocols are detailed for each tumor type below). Tissue quality was assessed based on visual examination and rapid pathology interpretation at the time of collection, and determined based on tumor content, necrosis, calcification, fat and hemorrhage.

For ovarian cancer ascites, ~300 ml was usually received from the hospital team within 1 h after being taken out of the body, and contained a vast majority of non-malignant (mainly immune) cells. Hence, all ascites samples were subjected to CD45^+^ cell depletion (below) to enrich for malignant cells.

For CLL, samples were generated from peripheral blood mononuclear cells isolated using density centrifugation (Ficoll-Paque) and stored in freezing medium (FBS + 10% DMSO) in liquid nitrogen until processing.

For O-PDX of neuroblastoma samples *Foxn1*^−*/*−^ nude mice (Charles River Laboratories) were orthotopically injected via ultrasound-guided para-adrenal injection with cells derived from a patient MYCN-amplified neuroblastoma (available as sample SJNBL046_X1 through the Childhood Solid Tumor Network)^19,20^. A portion of O-PDX tumor was flash-frozen for future snRNA-Seq, while the remainder underwent dissociation as described below.

#### Preservation of tissue for snRNA-Seq

For those samples that we prospectively collected for snRNA-Seq (neuroblastoma HTAPP-244-SMP-451 and HTAPP-656-SMP-3481), freezing of tumor samples was performed as quickly as possible after sample collection using a standard biobanking technique and the dates when samples were frozen were recorded. (Other samples were obtained from tissue banks with a limited record on how they were frozen, which is a typical scenario.) Samples were placed in cryo-tubes without any liquid. Complete removal of liquid from the sample was accomplished by gently wiping it (not patting, as this would damage the tissue) on the side of the container, before placing in the cryotube. The tubes were then covered in dry ice and transferred to −80 °C for long-term storage.

The other frozen samples from snRNA-Seq were obtained from tissue banks as follows: ovarian optimal cutting temperature compound (OCT)-frozen archival samples were obtained from the DFCI Gynecology Oncology Tissue Bank; sarcoma snap-frozen samples were obtained from the Boston Children’s Hospital Tissue Bank; pediatric snap-frozen glioma samples were obtained from the St Jude Children’s Research Hospital Biorepository; neuroblastoma snap-frozen samples were obtained from the St Jude Children’s Research Hospital Biorepository and the Boston Children’s Hospital Precision Link Biobank for Health Discovery; MBC OCT-frozen samples were obtained from the Center for Cancer Precision Medicine Bank; snap-frozen melanoma samples were obtained through the laboratory of Dr C. Yoon at BWH.

#### Dissociation workflow from fresh solid tumor samples to a single-cell suspension for scRNA-Seq

##### MBC, NSCLC (protocols PDEC and LE), ovarian cancer solid tumor and neuroblastoma workflows

Fresh tissue dissociation of MBC, NSCLC (protocols PDEC and LE), ovarian cancer solid tumor and neuroblastoma were performed using a similar workflow (Fig. 2a), with different components of the dissociation mixture for each tumor type, as described in the next section.

Samples were transferred from interventional radiology (biopsies) or the operating room (resections) in DMEM (MBC), RPMI (NSCLC) or RPMI with HEPES (ovarian cancer and neuroblastoma) medium. On arrival at the laboratory, the sample was washed in cold PBS and transferred into either a 2 ml Eppendorf tube containing dissociation mixture (for biopsies) or a 5 ml Eppendorf tube containing 3 ml dissociation mixture (for resections). Next, the sample was minced in the Eppendorf tube using spring scissors (Fine Science Tools, cat. no. 15514-12) into fragments under ~0.4 mm, and incubated at 37 °C, while rotating horizontally at ~14 r.p.m., for 10 min. After 10 min, the sample was pipetted 20 times with a 1 ml pipette tip at room temperature and placed back into incubation with rotation for an additional 10 min. The sample was pipetted again 20 times using a 1 ml pipette tip, transferred to a 1.7 ml Eppendorf tube and centrifuged at 300–580*g* for 4–7 min at 4 °C. The supernatant was removed and the pellet was resuspended in 200–500 µl of ACK (ammonium-chloride-potassium) RBC lysis buffer (Thermo Fisher Scientific, A1049201). The ACK volume added depended on the size of the pellet; while pellet size is hard to quantify, we suggest adding ~100 µl ACK lysis buffer per 100,000 cells, with a minimum volume of 200 µl. The sample was incubated in ACK RBC lysis buffer for 1 min on ice, followed by the addition of cold PBS at twice the volume of the ACK. The cells were pelleted by a short centrifugation for 8 s at 4 °C using the short spin setting with centrifugal force ramping up to, but not exceeding, 11,000*g*. The supernatant was removed. The pellet color was assessed; if RBCs remained (pellet color pink or red), the ACK step was repeated up to two additional times. To remove cell clumps in the MBC protocol (or sample), the pellet was resuspended in 100 µl of TrypLE (Life Technologies, cat. no. 12604013) and incubated while constantly pipetting at room temperature for 1 min with a 200 µl pipette tip. TrypLE was inactivated by adding 200 µl of cold RPMI 1640 with 10% FBS. The cells were pelleted using short centrifugation as described above. The pellet was resuspended in 50 µl of 0.4% BSA (Ambion, cat. no. AM2616) in PBS. To assess the single-cell suspension, viability and cell count, 5 µl of Trypan blue (Thermo Fisher Scientific, cat. no. T10282) was mixed with 5 µl of the sample and loaded onto an INCYTO C-Chip Disposable Hemocytometer, Neubauer Improved (VWR, cat. no. 82030-468). The cell concentration was adjusted if necessary to a range of 200–2,000 cells per µl. A total of 8,000 cells were loaded into each channel of the 10x Genomics Single-Cell Chromium Controller. Due to differences between clinical samples, some steps may need to be repeated or adjusted; for a general overview of guidelines see Fig. 2a.

##### NSCLC-C4 protocol workflow

A similar workflow was used for protocol NSCLC-C4 with the following modifications. Following mechanical chopping as above, sample was dissociated for 15 min in a 15 ml falcon tube, with a gentle vortex every 5 min, followed by filtration through a 70 µm filter, and washed with 20 ml of ice-cold PBS and centrifuged at 580*g* for 5 min. RBC lysis was performed similarly to the above workflow by resuspending the pellet in 1 ml ACK lysis buffer with incubation on ice for 1 min. A 20 ml volume of ice-cold PBS was added to quench the ACK lysis buffer, followed by filtration through a 70 µm filter, and centrifugation at 580*g* for 5 min. Sample NSCLC14 was further cleaned using a Viahance dead-cell removal kit (BioPAL, cat. no. CP-50VQ02) according to the manufacturer’s instructions. Cells were then resuspended in M199 and loaded on the 10x Genomics Single-Cell Chromium Controller as described above.

##### GBM workflow

All steps were completed on ice. Each sample was minced thoroughly in a Petri dish, 4 ml HBSS was added (Life Technologies, cat. no. 14175095), then the sample was transferred to 15 ml tubes and centrifuged at 1,000 r.p.m. for 2 min. After centrifugation, supernatant was removed, pre-heated dissociation mixture was added, and the sample was incubated while shaking at 37 °C for 15 min. Sample was pipetted up–down 20 times, incubated at 37 °C for an additional 15 min, and pipetted again. After dissociation, the sample was filtered through a 100 μm cell strainer (Fisher Scientific, cat. no. 22–363–549) into a 50 ml tube. We recommend keeping any tissue fragments left in the cell strainer, as they can be reprocessed with the same protocol if initial cell recovery is low. The filtrate was centrifuged at 1,000 r.p.m. for 3 min, and the supernatant was removed. If the pellet was bloody, RBC removal was performed when needed using Lympholyte H (Cedarlane, cat. no. CL5015) or RBC Lysis Solution (10×) (Miltenyi Biotec, cat. no. 130-094-183). The pellet was washed with 10 ml of cold PBS/1% BSA, transferred to a 15 ml tube and centrifuged at 1,200 r.p.m. for 3 min. Supernatant was removed and the pellet was resuspended in 0.4% BSA in PBS. The single-cell suspension was visualized, counted and loaded on the 10x Genomics Single-Cell Chromium Controller as described above.

#### Dissociation mixtures for different tumor types

Dissociation mixtures were prepared ~5–10 min before sample processing from frozen aliquoted stocks, as follows.

##### MBC LD protocol

A 950 µl volume of RPMI 1640 (Thermo Fisher Scientific, cat. no. 11875093) was used with 10 µl of 10 mg ml^−1^ DNAse I (Sigma Aldrich, cat. no. 11284932001) to a final concentration of 100 µg ml^−1^, and 40 µl of 2.5 mg ml^−1^ Liberase TM (Sigma Aldrich, cat. no. 5401127001).

##### Ovarian cancer resection MHTD kit

The dissociation mixture was based on the Miltenyi Human Tumor Dissociation Kit (Miltenyi Biotec, cat. no. 130-095-929). Before starting, enzymes H, R and A were resuspended according to the manufacturer’s instructions. Dissociation mix containing 2.2 ml RPMI, 100 µl enzyme H, 50 µl enzyme R and 12.5 µl enzyme A was prepared immediately before use.

##### Neuroblastoma NB-C4 protocol

Medium 199 with Hanks balanced salts buffer (Thermo Fisher Scientific) was used with 100 µg ml^−1^ of DNAse I (Millipore Sigma, cat. no. 11284932001) and 100 µg ml^−1^ collagenase IV (Worthington, cat. no. LS004186).

##### O-PDX neuroblastoma

A papain kit, the Worthington Papain Dissociation System (cat. no. LK003150), was used. Dissociation was performed according to the manufacturer’s instructions, with deviation of the dissociation duration, which was shortened to 15 min.

##### NSCLC PDEC protocol

We used 2692 µl HBSS (Thermo Fisher Scientific, cat. no. 14170112), 187.5 µl of 20 mg ml^−1^ pronase (Sigma Aldrich, cat. no. 10165921001) to a final concentration of 1,250 µg ml^−1^, 27.6 µl of 1 mg ml^−1^ elastase (Thermo Fisher Scientific, cat. no. NC9301601) to a final concentration of 9.2 µg ml^−1^, 30 µl of 10 mg ml^−1^ DNase I (Sigma Aldrich, cat. no. 11284932001) to a final concentration of 100 µg ml^−1^, 30 µl of 10 mg ml^−1^ Dispase (Sigma Aldrich, cat. no. 4942078001) to a final concentration of 100 µg ml^−1^, 30 µl of 150 mg ml^−1^ collagenase A (Sigma Aldrich, cat. no. 10103578001) to a final concentration of 1,500 µg ml^−1^ and 3 µl of 100 mg ml^−1^ collagenase IV (Thermo Fisher Scientific, cat. no. NC9836075) to a final concentration of 100 µg ml^−1^.

##### NSCLC LE protocol

We used 4.7 ml RPMI 1640 (Thermo Fisher Scientific, cat. no. 11875093), 200 µl of 2.5 mg ml^−1^ Liberase TM (Millipore Sigma, cat. no. 5401119001) to a final concentration of 100 µg ml^−1^, 50 µl of 10 mg ml^−1^ DNase I (Sigma Aldrich, cat. no. 11284932001) to a final concentration of 100 µg ml^−1^ and 46 µl of 1 mg ml^−1^ elastase (Thermo Fisher Scientific, cat. no. NC9301601) to a final concentration of 9.2 µg ml^−1^.

##### NSCLC-C4 protocol

M199 (5 ml) was used with DNase 1 (final concentration of 10 µg ml) and collagenase IV (final concentration of 100 µg ml^−1^).

##### GBM BTD kit

A Brain Tumor Dissociation Kit (P) (Miltenyi Biotech, cat. no. 130-095-942) was used with 4 ml buffer X, 40 µl buffer Y, 50 µl enzyme N and 20 µl enzyme A.

#### Processing of non-solid tumor samples for scRNA-Seq

##### CLL

Frozen (cryopreserved) cells were thawed in 10 ml RPMI, pelleted and washed with an additional 10 ml RPMI. Live cells were sorted using the MoFlo Astrios EQ Cell Sorter and 8,000 cells were loaded on one channel of the 10x Genomics Single-Cell Chromium Controller. Remaining cells were pelleted by short centrifugation, the supernatant was discarded and the pellet was frozen on dry ice and stored at −80 °C.

##### Ovarian cancer ascites

Ascites samples without spheres were selected and delivered in four 50 ml conical tubes, for a total of 200 ml of fluid. Tubes were spun down at 580*g* for 5 min in a 4 °C pre-cooled centrifuge and supernatants were aspirated.

Pellets were resuspended in 5 ml cold ACK lysing buffer and combined from all tubes at this step. ACK lysis was done on ice for 3 min, and quenched by adding 10 ml of cold PBS, followed by centrifugation at 580*g* for 5 min at 4 °C. Pellet color was assessed; if it was pink or red, revealing a significant portion of erythrocytes, ACK treatment steps were repeated as needed for two additional times, at most. Post ACK treatment, the pellet was resuspended in 20 ml cold PBS, filtered through a 70 µm cell strainer into a 50 ml conical tube, and the filter was washed with additional 20 ml cold PBS to recover as many cells as possible. The sample was then centrifuged at 580*g* for 5 min at 4 °C. To reduce the fraction of immune cells in the sample, CD45^+^ cell depletion was performed using the MACS CD45 depletion protocol described below.

#### Depletion of CD45^+^ cells for scRNA-Seq

Depletion of CD45^+^ cells in ovarian cancer ascites samples and NSCLC samples was performed using CD45 MicroBeads (Miltenyi Biotec, cat. no. 130-045-801) according to the manufacturer’s protocol. Briefly, following filtration of the ovarian cells from ascites or dissociation of NSCLC tissue samples, cells were counted. The single-cell suspension was centrifuged at 500*g* for 4 min at 4 °C. The supernatant was removed and the pellet was resuspended in 80 µl of MACS buffer (PBS supplemented with 0.5% BSA, and 2 mM EDTA) per 10^6^ cells. MACS CD45 microbeads were added to the cell suspension (20 µl per 10 million cells). The cells were incubated on ice for 15 min. During incubation, the column (MS for NSCLC and LS for ovarian ascites) was prepared by attaching the column to a MidiMACS separator and rinsing the column with 3 ml MACS buffer. Following incubation, the cells and bead conjugate were washed with 900 µl MACS buffer per 10 million cells. The cells were centrifuged at 500*g* for 4 min at 4 °C. The supernatant was removed and the pellet was resuspended in 500 µl MACS buffer. The cell suspension was transferred to the column and the effluent was collected (CD45^−^ fraction). The column was washed three times with 3 ml MACS buffer. The CD45^−^ fraction was centrifuged at 500*g* for 4 min at 4 °C. In the ascites sample, bead attachment and column separation can be repeated to increase the number of tumor and stromal cells relative to immune cells. The pellet was resuspended in 50 µl of 0.4% BSA (Ambion, cat. no. AM2616) in PBS. Cells were counted by mixing 5 µl of Trypan blue (Thermo Fisher Scientific, cat. no. T10282) with 5 µl of the sample and loaded on INCYTO C-Chip Disposable Hemocytometer, Neubauer Improved (VWR, cat. no. 82030-468). The cell concentration was adjusted if necessary to a range of 200–2,000 cells per µl. A total of 8,000 cells were loaded into each channel of the 10x Genomics Single-Cell Chromium Controller.

#### Flow cytometry analysis

For flow cytometry analysis of CD45^+^ depletion in the ovarian cancer ascites sample, cells were resuspended in PBS complemented with 2% FBS and stained with FITC anti-human CD45 antibody (BioLegend, cat. no. 304006CD45; 1:200 dilution), PE anti-human EpCAM antibody (Miltenyi Biotech, cat. no. 130-113-264; 1:50 dilution), APC anti-human CD14 (BioLegend, cat. no. 367118, clone 63D3; 1:20 dilution) and PE-cy7 anti-human CD24 (BioLegend, cat. no. 311120, clone ML5; 1:20 dilution) for 20 min, and with 7-AAD (Invitrogen, cat. no. A1310; 1:200 dilution) for 5 min. The same cells were also used for single-stain and unstained controls to perform compensation and adjust gating. Analysis was performed on a BD LSRFortessa cell analyzer with BD FACSDiva Software Version 8.0.1 and plots were generated with FlowJo Version 10.5.3. Gating for CD45 and EpCAM was performed as described in Extended Data Fig. 4c. CD24 and CD14 antibodies were included in the antibody panel for FACS analysis to provide additional information and better inform scRNA-Seq. Specifically, expression of CD24 on tumor cells has been shown to relate to ovarian cancer invasiveness and expression of CD14 identifies monocytes/macrophages.

#### ST-based buffers for snRNA-Seq

A 2× stock of salt-Tris solution (ST buffer) containing 292 mM NaCl (Thermo Fisher Scientific, cat. no. AM9759), 20 mM Tris-HCl pH 7.5 (Thermo Fisher Scientific, cat. no. 15567027), 2 mM CaCl_2_ (VWR International Ltd, cat. no. 97062-820) and 42 mM MgCl_2_ (Sigma Aldrich, cat. no. M1028) in ultrapure water was made and used to prepare three buffers: for CST, 1 ml of 2× ST buffer, 980 µl of 1% CHAPS (Millipore, cat. no. 220201), 10 µl of 2% BSA (New England BioLabs, cat. no. B9000S) and 10 µl of nuclease-free water; for TST, 1 ml of 2× ST buffer, 60 µl of 1% Tween-20 (Sigma Aldrich, cat. no. P-7949), 10 µl of 2% BSA (New England Biolabs, cat. no. B9000S) and 930 µl of nuclease-free water; for NST, 1 ml of 2× ST buffer, 40 µl of 10% Nonidet P40 Substitute (Fisher Scientific, cat. no. AAJ19628AP), 10 µl of 2% BSA (NEB) and 950 µl of nuclease-free water. 1× ST buffer was prepared by dilution 2× ST with ultrapure water (Thermo Fisher Scientific cat. no. 10977023) in a ratio of 1:1.

#### Nucleus isolation from frozen samples for snRNA-Seq

On dry ice, tissue was split and subjected to one of three ST-based nucleus isolation protocols^11^ and the EZ nucleus isolation buffer^8^, as detailed in the following.

##### Nucleus isolation workflow for ST-based buffers

On ice, a piece of frozen tumor tissue was placed into a well of a 6-well plate (Stem Cell Technologies, cat. no. 38015) with 1 ml of CST, TST or NST buffer. For samples frozen in OCT, an additional step of removing the surrounding OCT and washing any residual OCT from the sample with PBS was performed in a 10 cm Petri dish. Tissue was then chopped using Noyes Spring Scissors (Fine Science Tools, cat. no. 15514-12) for 10 min on ice. For cell pellets, such as for CLL frozen cells, sample was pipetted in the buffer on ice, instead of chopping. The homogenized solution was then filtered through a 40 µm Falcon cell strainer (Thermo Fisher Scientific, cat. no. 08-771-1). An additional 1 ml of the detergent buffer solution was used to wash the well and filter. The volume was brought up to 5 ml with 3 ml of 1× ST buffer. The sample was then transferred to a 15 ml conical tube and centrifuged at 4 °C for 5 min at 500*g* in a swinging bucket centrifuge. The pellet was resuspended in 1× ST buffer. Resuspension volume was dependent on the size of the pellet, usually within the range of 100–200 µl. The nucleus solution was then filtered through a 35 µm Falcon cell strainer (Corning, cat. no. 352235). Nuclei were counted using a C-chip disposable hemocytometer (VWR, cat. no. 82030-468). Either 10,000 or 8,000 nuclei (V2 or V3 10x Genomics, respectively) of the single-nucleus suspension were loaded onto the Chromium Chips for the Chromium Single Cell 3′ Library (V2, PN-120233; V3, PN-1000075) according to the manufacturer’s recommendations (10x Genomics).

##### Nucleus isolation workflow using EZ lysis buffer

Nucleus isolation was done as previously described^8^. Briefly, tissue samples were cut into pieces <0.5 cm and homogenized using a glass Dounce tissue grinder (Sigma, cat. no. D8938). The tissue was homogenized 25 times with pestle A and 25 times with pestle B in 2 ml of ice-cold nuclei EZ lysis buffer. The sample was then incubated on ice for 5 min, with an additional 3 ml of cold EZ lysis buffer. Nuclei were centrifuged at 500*g* for 5 min at 4 °C, washed with 5 ml ice-cold EZ lysis buffer and incubated on ice for 5 min. After centrifugation, the nucleus pellet was washed with 5 ml nuclei suspension buffer (NSB; consisting of 1× PBS, 0.01% BSA and 0.1% RNase inhibitor (Clontech, cat. no. 2313A)). Isolated nuclei were resuspended in 2 ml NSB, filtered through a 35 μm cell strainer (Corning-Falcon, cat. no. 352235) and counted. A final concentration of 1,000 nuclei per µl was used for loading on a 10x channel.

#### Droplet-based sc/snRNA-Seq

For V2 10x technology, either 8,000 single cells or 10,000 single nuclei were loaded into each channel of a Chromium single-cell 3′ Chip. For V3 10x technology, 8,000 single cells and 8,000 single nuclei were loaded. Single cells/nuclei were partitioned into droplets with gel beads in the Chromium Controller. After emulsions were formed, barcoded reverse transcription of RNA took place. This was followed by cDNA amplification, fragmentation and adapter and sample index attachment, all according to the manufacturer’s recommendations. Libraries from four 10x channels were pooled together and sequenced on one lane of an Illumina HiSeq X, or on one flow cell of a NextSeq, with paired end reads as follows: read 1, 26 nt; read 2, 55 nt; index 1, 8 nt; index 2, 0 nt.

### Computational methods

#### scRNA-Seq data processing

We used Cell Ranger mkfastq (v2.0 and v3.0) (10x Genomics) to generate demultiplexed FASTQ files from the raw sequencing reads. We aligned these reads to the human GRCh38 genome and quantified gene counts as UMIs using Cell Ranger count (v2.0 and v3.0) (10x Genomics). For snRNA-Seq reads, we counted reads mapping to introns as well as exons, as this results in a greater number of genes detected per nucleus, more nuclei passing quality control and better cell type identification, as previously described^38^. To count introns during read mapping, we followed the approach described at https://support.10xgenomics.com/single-cell-gene-expression/software/pipelines/latest/advanced/references. Briefly, we built a ‘pre-mRNA’ human GRCh38 reference using Cell Ranger mkref (v3.0) (10x Genomics) and a modified gene transfer format (GTF) file, where, for each transcript, the feature type had been changed from transcript to exon. The starting GTF files came from refdata-cellranger-GRCh38-1.2.0.tar.gz or refdata-cellranger-GRCh38-3.0.0.tar.gz, and are available for download at https://support.10xgenomics.com/single-cell-gene-expression/software/downloads/3.0.

To downsample sequencing reads or gene counts (UMIs) when comparing protocols, we used downsampleReads and downsampleMatrix, respectively, from the R package DropletUtils (v1.0.3 or higher)^12^. Reads were downsampled to match the protocol with the lowest number of total reads. After downsampling by total reads, we used write10xCounts from DropletUtils and a custom Python script to generate an HDF5 file for input into our analysis pipelines, as described in the sections that follow and in the ‘Code availability’ section.

#### QC of scRNA-Seq data

To maintain explicit control over all gene and cell quality control filters, in all our downstream analyses we used the raw feature-barcode matrix, rather than the filtered feature-barcode matrix generated by Cell Ranger. We removed low-quality cells by requiring each cell to have a minimal number of UMIs and genes detected. We used different thresholds depending on the experimental modality (single cell or single nucleus) and on the 10x kit (V2 or V3 chemistry). For single nucleus data, we retained nuclei with at least 200 genes and 400 UMIs detected by V2 chemistry and with at least 500 genes and 1,000 UMIs detected by V3 chemistry. For single-cell data, we retained cells with at least 500 genes and 1,000 UMIs detected by either V2 or V3 chemistry. For the V2–V3 comparison in HTAPP-951-SMP-4652 (Extended Data Fig. 9), we used the same thresholds for both chemistries: at least 200 genes and 400 UMIs detected. For both data types, we filtered out those cells or nuclei where >20% of UMIs came from mitochondrial genes. Finally, we normalized the total UMIs per cell or nucleus to 100,000 (CP100K) and log-transformed these values to report gene expression as *E* = log(CP100K + 1).

We reported the following QC metrics: number of total reads per library sample, sequencing saturation (fraction of reads originating from an already-observed UMI as reported by Cell Ranger count), total recovered cells or nuclei, number of reads per cell or nucleus, number of UMIs per cell or nucleus, number of genes detected per cell or nucleus, fraction of UMIs in a cell or nucleus aligned to mitochondrial genes, fraction of droplets estimated to contain only ambient RNA (‘empty drops’), fraction of cell or nucleus doublets, the number of detected cell types and the pattern of CNAs for malignant cells. For a subset of samples, we also calculated the number of cells or nuclei per detected cell type and the estimated level of ambient RNA in droplets containing cells.

We predicted droplets containing only ambient RNA and no cells using EmptyDrops (part of DropletUtils, v1.0.3 or higher), with the retain parameter set by the knee of the curve in the barcode rank plot (cell barcodes ranked by their total UMIs)^12^. We predicted potential doublets using Scrublet (v0.2) with expected_doublet_rate = 0.06 (ref. ^13^). We estimated the levels of ambient RNA using SoupX (v0.3.1)^18^ and a set of cell-type-specific marker genes (Supplementary Table 1). Importantly, we flagged the doublets and empty drops and retained them in our analysis, instead of immediately filtering them out. Droplets that appear to contain doublets or empty drops can arise from many different effects, such as cellular differentiation or insufficient sequencing, and by carrying them through the analysis, potential doublets or empty drops can be more clearly interpreted in the context of the full dataset.

#### Dimensionality reduction, clustering and visualization

For each tumor sample, we analyzed the filtered expression matrix to identify cell subsets, as previously described^39,40^. We chose highly variable genes with a *z*-score cutoff of 0.5 (ref. ^41^), centered and scaled the expression of each gene to have a mean of zero and standard deviation of one, and performed dimensionality reduction on the variable genes using principal component analysis. We used the top 50 principal components (PCs) as input to Louvain graph-based clustering, with the resolution parameter set to 1.3. For each cluster of cells, we identified cluster-specific differentially expressed genes using the following tests: an AUC classifier, Welch’s *t*-test and Fisher’s exact test. For tests that returned a *P* value, we controlled the false discovery rate at 5% with the Benjamini–Hochberg procedure^42^. We visualized gene expression and clustering results by embedding cells or nuclei profiles in a Uniform Manifold Approximation and Projection (UMAP)^43^ of the top 50 PCs, with min_dist = 0.5, spread = 1.0, number of neighbors = 15 and the Euclidean distance metric.

#### Annotating cell subsets

For each cell subset identified by clustering, we assigned a cell type from the malignant, parenchymal, stromal and immune compartments of the tumor microenvironment using a combination of differentially expressed genes, known gene signatures (Supplementary Table 1) and SingleR (v0.2.2)^44^, an automated annotation package. When running SingleR, only cell types assigned to 30 or more cells were considered. When scoring cells for the expression of known gene signatures, we used the AddModuleScore function in Seurat (v2.3.4)^24^. We note that overlapping expression programs between T cells and NK cells make these cell types sometimes more difficult to identify accurately. We did not distinguish macrophages, monocytes and dendritic cells, and annotated all of these as scoring for ‘macrophages’ signatures.

We identified the malignant cells by inferring chromosomal CNAs from the gene-expression data using inferCNV (v1.1.0)^45^. On a sample-by-sample basis, we used the immune and endothelial cells as a healthy reference to estimate CNAs in the malignant cells. We created the count matrix file and annotation file for inferCNV by randomly subsetting the counts data to sample at most 2,000 cells or nuclei. We created a gene ordering file from the human GRCh38 assembly, which contains the chromosomal start and end positions for each gene. To run inferCNV, we used a cutoff of 0.1 for the minimum average read counts per gene among reference cells or nuclei, clustered according to the annotated cell types, denoised our output, ran a hidden Markov model (HMM) to predict the CNA level, implemented inferCNV’s i6 HMM model, and requested eight threads for parallel steps.

#### Comparing sc- and snRNA-Seq data

To compare profiles between sc- and snRNA-Seq data collected from the same sample, we used a batch correction approach.

We performed batch correction using CCA as implemented in Seurat (v2.3.4)^24^. We selected 1,500 genes that were variable across both the cell and nucleus data, used those genes as input to RunCCA to compute the first 20 canonical components, and aligned the first 12 canonical components with AlignSubspace. The aligned canonical components represent a co-embedding of the cell and nucleus data, and we carried out clustering in this dimensionality-reduced space using FindClusters.

Following batch correction by CCA, we scored the dissociation signature from ref. ^25^ (from their Supplementary Table 5) on our matched cell/nuclei samples using the AddModuleScore function in Seurat (v2.3.4)^24^.

### Reporting Summary

Further information on research design is available in the Nature Research Reporting Summary linked to this Article.

## Online content

Any methods, additional references, Nature Research reporting summaries, source data, extended data, supplementary information, acknowledgements, peer review information; details of author contributions and competing interests; and statements of data and code availability are available at 10.1038/s41591-020-0844-1.

## Supplementary information

Reporting Summary

Supplementary Table 1 Supplementary Table 1

## Data Availability

All main and extended data figures have associated raw data. Raw data will be available in the controlled access repository dbGaP (https://www.ncbi.nlm.nih.gov/gap/), under dbGaP Study Accession phs001983.v1.p1. Raw data will also be available in the controlled access repository DUOS (https://duos.broadinstitute.org/), under DUOS Dataset IDs DUOS-000111, DUOS-000112, DUOS-000113 and DUOS-000114. The counts matrices and metadata for each sample will be publicly available in the Gene Expression Omnibus (https://www.ncbi.nlm.nih.gov/geo/) under data repository accession no. GSE140819. Finally, we provide a website that displays a comprehensive analysis summary for each sample tested (https://tumor-toolbox.broadinstitute.org).

## Code availability

We implemented all initial analysis steps, from FASTQ files to clustering cell subsets, in Cumulus^14^, which may be executed in both a cloud-based environment and locally. Pipelines were written in the Workflow Description Language (WDL) and run on Cromwell in the Terra Cloud platform (https://app.terra.bio/), and data were stored in Google Cloud Platform storage buckets. Cumulus workflows are publicly available at https://github.com/klarman-cell-observatory/Cumulus.

We built and ran an additional pipeline for subsequent QC steps, including detecting empty drops and doublets, annotating cell subsets, evaluating cell type specific QCs and inferring CNAs. This pipeline was implemented in R (v3.5 or higher) by converting the single-cell AnnData objects from Cumulus into Seurat objects, and was used to compare and evaluate processing protocols. Our analysis pipeline, including complete example analysis for one scRNA-Seq sample and one snRNA-Seq sample, will be made publicly available at https://github.com/klarman-cell-observatory/HTAPP-Pipelines.
