# Author Correction: A single-cell and single-nucleus RNA-Seq toolbox for fresh and frozen human tumors

**DOI:** 10.1038/s41591-020-0976-3

**Published:** 2020-06-25

**Authors:** Michal Slyper, Caroline B. M. Porter, Orr Ashenberg, Julia Waldman, Eugene Drokhlyansky, Isaac Wakiro, Christopher Smillie, Gabriela Smith-Rosario, Jingyi Wu, Danielle Dionne, Sébastien Vigneau, Judit Jané-Valbuena, Timothy L. Tickle, Sara Napolitano, Mei-Ju Su, Anand G. Patel, Asa Karlstrom, Simon Gritsch, Masashi Nomura, Avinash Waghray, Satyen H. Gohil, Alexander M. Tsankov, Livnat Jerby-Arnon, Ofir Cohen, Johanna Klughammer, Yanay Rosen, Joshua Gould, Lan Nguyen, Matan Hofree, Peter J. Tramontozzi, Bo Li, Catherine J. Wu, Benjamin Izar, Rizwan Haq, F. Stephen Hodi, Charles H. Yoon, Aaron N. Hata, Suzanne J. Baker, Mario L. Suvà, Raphael Bueno, Elizabeth H. Stover, Michael R. Clay, Michael A. Dyer, Natalie B. Collins, Ursula A. Matulonis, Nikhil Wagle, Bruce E. Johnson, Asaf Rotem, Orit Rozenblatt-Rosen, Aviv Regev

**Affiliations:** 1 Klarman Cell Observatory, Broad Institute of Harvard and MIT, Cambridge, MA USA; 2 Broad Institute of Harvard and MIT, Cambridge, MA USA; 3 Department of Medical Oncology, Dana-Farber Cancer Institute, Boston, MA USA; 4 Center for Cancer Precision Medicine of Dana-Farber Cancer Institute, Boston, MA USA; 5 Department of Developmental Neurobiology, St Jude Children’s Research Hospital, Memphis, TN USA; 6 Department of Oncology, St Jude Children’s Research Hospital, Memphis, TN USA; 7 Department of Pathology, Massachusetts General Hospital and Harvard Medical School, Boston, MA USA; 8 Center for Cancer Research, Massachusetts General Hospital and Harvard Medical School, Boston, MA USA; 9 Center for Regenerative Medicine, Massachusetts General Hospital, Boston, MA USA; 10 Division of Thoracic Surgery, Brigham and Women’s Hospital, Boston, MA USA; 11 Center for Immunology and Inflammatory Diseases, Division of Rheumatology, Allergy, and Immunology, Massachusetts General Hospital and Harvard Medical School, Boston, MA USA; 12 Department of Internal Medicine, Brigham and Women’s Hospital, Boston, MA USA; 13 Harvard Medical School, Boston, MA USA; 14 Laboratory for Systems Pharmacology, Harvard Medical School, Boston, MA USA; 15 Center for Immunology and Virology, Dana-Farber Cancer Institute, Boston, MA USA; 16 Ludwig Center for Cancer Research at Harvard, Boston, MA USA; 17 Melanoma Disease Center, Dana-Farber Cancer Institute, Boston, MA USA; 18 Department of Surgical Oncology, Brigham and Women’s Hospital, Boston, MA USA; 19 Department of Medicine, Massachusetts General Hospital and Harvard Medical School, Boston, MA USA; 20 Department of Pathology, St Jude Children’s Research Hospital, Memphis, TN USA; 21 Howard Hughes Medical Institute, Chevy Chase, MD USA; 22 Department of Pediatric Oncology, Dana-Farber Cancer Institute, Boston, MA USA; 23 Division of Pediatric Hematology and Oncology, Boston Children’s Hospital, Boston, MA USA; 24 Koch Institute for Integrative Cancer Research, Department of Biology, Massachusetts Institute of Technology, Cambridge, MA USA; 25 Present Address: Icahn School of Medicine at Mount Sinai, New York, NY USA; 26 Present Address: Columbia Center for Translational Immunology and Division of Hematology and Oncology, Columbia University Medical Center, New York, NY USA

**Keywords:** Gene expression analysis, Cancer, Computational biology and bioinformatics, Biological techniques, Genomic analysis

Correction to: *Nature Medicine* 10.1038/s41591-020-0844-1, published online 11 May 2020.

In the version of this article initially published, two concentrations were incorrect (“3 ml of 100 μg ml^−1^ collagenase IV” and “final concentration of 1,250 μg ml^−1^”) at the end of the final sentence of the Methods subsection ‘NSCLC PDEC protocol’. The correct statement is as follows: “...3 ml of 100 mg ml^−1^ collagenase IV (Thermo Fisher Scientific, cat. no. NC9836075) to a final concentration of 100 μg ml^−1^.” The error has been corrected in the HTML and PDF versions of the article.

