# Supplementary material for: A single-cell and single-nucleus RNA-Seq toolbox for fresh and frozen human tumors
Source: Nat Med. 2020 May 11;26(5):792–802. doi: 10.1038/s41591-020-0844-1 (PMC7220853; doi:10.1038/s41591-020-0844-1)
Supplement: Supplementary file 1 — Reporting Summary [file 41591_2020_844_MOESM1_ESM.pdf]

nature research

Corresponding author(s): Aviv Regev and Orit Rozenblatt-Rosen

Last updated by author(s): 03/04/2020

Reporting Summary

Nature Research wishes to improve the reproducibility of the work that we publish. This form provides structure for consistency and transparency in reporting. For further information on Nature Research policies, see Authors & Referees and the Editorial Policy Checklist.

Statistics

For all statistical analyses, confirm that the following items are present in the figure legend, table legend, main text, or Methods section.

n/a	Confirmed	
☐	☒	The exact sample size (n) for each experimental group/condition, given as a discrete number and unit of measurement
☐	☒	A statement on whether measurements were taken from distinct samples or whether the same sample was measured repeatedly
☐	☒	The statistical test(s) used AND whether they are one- or two-sided<br>*Only common tests should be described solely by name; describe more complex techniques in the Methods section.*
☒	☐	A description of all covariates tested
☐	☒	A description of any assumptions or corrections, such as tests of normality and adjustment for multiple comparisons
☐	☒	A full description of the statistical parameters including central tendency (e.g. means) or other basic estimates (e.g. regression coefficient) AND variation (e.g. standard deviation) or associated estimates of uncertainty (e.g. confidence intervals)
☐	☒	For null hypothesis testing, the test statistic (e.g. F, t, r) with confidence intervals, effect sizes, degrees of freedom and P value noted<br>*Give P values as exact values whenever suitable.*
☒	☐	For Bayesian analysis, information on the choice of priors and Markov chain Monte Carlo settings
☒	☐	For hierarchical and complex designs, identification of the appropriate level for tests and full reporting of outcomes
☒	☐	Estimates of effect sizes (e.g. Cohen's d, Pearson's r), indicating how they were calculated

Our web collection on statistics for biologists contains articles on many of the points above.

Software and code

Policy information about availability of computer code

Data collection	We did not use any software for data collection.
Data analysis	1) Cell Ranger mkfastq (v2.0 and v3.0) (10x Genomics) to generate demultiplexed FASTQ files from the raw sequencing reads<br>2) Cell Ranger count (v2.0 and v3.0) (10x Genomics) to align reads and quantify gene counts as UMIs<br>3) Cell Ranger mkref (v3.0) (10x Genomics) to build a custom reference<br>4) R (v3.5 or higher) for gene expression analyses<br>5) RStudio (v1.2.1335) for running R analyses<br>6) Python (v3.7) for gene expression analyses<br>7) DropletUtils (v1.0.3 or higher, R package, http://bioconductor.org/packages/release/bioc/html/DropletUtils.html) to estimate droplets containing only ambient RNA<br>8) Scrublet (v0.2, Python package, https://github.com/AllonKleinLab/scrublet) to estimate droplets contain doublets<br>9) SoupX (v0.3.1, R package, https://github.com/constantAmateur/SoupX) to estimate ambient RNA in droplets that also contain cells<br>10) SingleR (v0.2.2, R package, https://github.com/dviraran/SingleR) for automated draft annotation<br>11) Seurat (v2.3.4, R package, https://satijalab.org/seurat/install.html) as a framework for additional quality control steps, cell-subset annotation, and cell/nuclei batch correction<br>12) inferCNV (v1.1.0, https://github.com/broadinstitute/infercnv) for inferring chromosomal copy number aberrations (CNAs) from the gene-expression data<br>13) Cumulus (https://github.com/klarman-cell-observatory/Cumulus), developed by Bo Li and his colleagues, is used to perform all major single-cell and single-nucleus RNA-Seq data analysis<br>14) BD FACSDiva Software (v8.0.1) for flow cytometry analysis.<br>15) FlowJo (v10.5.3) for flow cytometry plotting

For manuscripts utilizing custom algorithms or software that are central to the research but not yet described in published literature, software must be made available to editors/reviewers. We strongly encourage code deposition in a community repository (e.g. GitHub). See the Nature Research guidelines for submitting code & software for further information.

Data

Policy information about availability of data

All manuscripts must include a data availability statement. This statement should provide the following information, where applicable:

- Accession codes, unique identifiers, or web links for publicly available datasets
- A list of figures that have associated raw data
- A description of any restrictions on data availability

All main and Extended Data figures have associated raw data. Raw data will be available in the controlled access repository dbGaP (https://www.ncbi.nlm.nih.gov/gap/), under the dbGaP Study Accession phs001983.v1.p1; raw data will also be available in the controlled access repository DUOS (https://duos.broadinstitute.org/), under the following DUOS Dataset IDs: DUOS-000111, DUOS-000112, DUOS-000113, and DUOS-000114. The counts matrices and metadata for each sample will be publicly available in Gene Expression Omnibus (GEO, https://www.ncbi.nlm.nih.gov/geo/) under data repository accession no. GSE140819. Finally, we provide a website that displays a comprehensive analysis summary for each sample tested (https://tumor-toolbox.broadinstitute.org).

Field-specific reporting

Please select the one below that is the best fit for your research. If you are not sure, read the appropriate sections before making your selection.

☒ Life sciences ☐ Behavioural & social sciences ☐ Ecological, evolutionary & environmental sciences

For a reference copy of the document with all sections, see nature.com/documents/nr-reporting-summary-flat.pdf

Life sciences study design

All studies must disclose on these points even when the disclosure is negative.

Sample size	For each sample, an input of 8,000 single cells or 8,000-10,000 single nuclei were loaded into each channel of the 10x Genomics Single-Cell Chromium Controller. These loading values were chosen to balance the probability of forming doublets with the goal of having maximal cell recovery and sufficient cell/nuclei recovery to reveal the heterogeneous landscape of the tumors.
Data exclusions	We removed low quality cells by requiring each cell to have a minimal number of UMIs and genes detected. We used different thresholds depending on the experimental modality (single cell or single nucleus) and on the 10x kit (V2 or V3 chemistry). For single nucleus data, we retained nuclei with at least 200 genes and 400 UMIs detected by V2 chemistry and with at least 500 genes and 1,000 UMIs detected by V3 chemistry. For single cell data, we retained cells with at least 500 genes and 1,000 UMIs detected by either V2 or V3 chemistry. For the V2-V3 comparison in HTAPP-951-SMP-4652 (Extended Data Fig. 9), we used the same thresholds for both chemistries: at least 200 genes and 400 UMIs detected. For both data types, we filtered out those cells or nuclei where >20% of UMIs came from mitochondrial genes.
Replication	Each biological sample is unique to a patient due to tumor heterogeneity, and furthermore, tissue samples from the same patient tumor may have intra-tumor heterogeneity. All single-cell dissociation protocols and single-nuclei isolation methods that we recommend were tested on more than one patient tumor sample (biological replicate) and protocol performance was consistent across the different samples tested. For under-performing protocols, we generally do not include replicate samples.
Randomization	We do not have experimental groups.
Blinding	We do not have experimental groups.

Reporting for specific materials, systems and methods

We require information from authors about some types of materials, experimental systems and methods used in many studies. Here, indicate whether each material, system or method listed is relevant to your study. If you are not sure if a list item applies to your research, read the appropriate section before selecting a response.

Materials & experimental systems

n/a	Involved in the study	
☐	☒	Antibodies
☒	☐	Eukaryotic cell lines
☒	☐	Palaeontology
☐	☒	Animals and other organisms
☐	☒	Human research participants
☒	☐	Clinical data

Methods

n/a	Involved in the study	
☒	☐	ChIP-seq
☐	☒	Flow cytometry
☒	☐	MRI-based neuroimaging

Antibodies

Antibodies used	1) FITC anti-human CD45 Antibody, BioLegend #304006, https://www.biolegend.com/en-us/products/fitc-anti-human-cd45-

antibody-707, Clone HI30, Lot #B226081, used at 1:200 dilution
2) CD45 MicroBeads, human, Miltenyi # 130-045-801, https://www.miltenyibiotec.com/US-en/products/macs-cell-separation/cell-separation-reagents/microbeads-and-isolation-kits/tumor-cells/cd45-microbeads-human.html
3) CD326 (EpCAM)-PE, human, Miltenyi Biotech #130-113-264, https://www.miltenyibiotec.com/US-en/products/macs-flow-cytometry/antibodies/primary-antibodies/cd326-epcam-antibodies-human-hea-125-1-50.html#pe:for-100-tests, Clone HEA-125, Lot #5190328519, used at 1:50 dilution
4) APC anti-human CD14, BioLegend #367118, https://www.biolegend.com/nl-nl/products/apc-anti-human-cd14-antibody-12901, Clone 63D3, Lot #B262993, used at 1:20 dilution
5) PE-cy7 anti-human CD24, BioLegend #311120, https://www.biolegend.com/nl-nl/products/pe-cy7-anti-human-cd24-antibody-6126, Clone ML5, Lot #B226384, used at 1:20 dilution

Validation

All of the antibodies used in this study were validated for use in human specimens by the manufacturers, as indicated below:

FITC anti-human CD45 Antibody, BioLegend #304006:
Application: FC - Quality tested (FC: Flow cytometric analysis of antibody surface-stained cells.)
Recommended Usage: Each lot of this antibody is quality control tested by immunofluorescent staining with flow cytometric analysis.

CD326 (EpCAM)-PE, human, Miltenyi Biotech # 130-113-264:
Peripheral blood leukocytes mixed with cells from a breast cancer cell line (SK-BR-3) were stained with CD326 (EpCAM) antibodies and analyzed by flow cytometry using the MACSQuant® Analyzer.

APC anti-human CD14, BioLegend #367118:
Application: FC - Quality tested (FC: Flow cytometric analysis of antibody surface-stained cells.)
Recommended Usage: Each lot of this antibody is quality control tested by immunofluorescent staining with flow cytometric analysis.

PE-cy7 anti-human CD24, BioLegend #311120:
Application: FC - Quality tested (FC: Flow cytometric analysis of antibody surface-stained cells.)
Recommended Usage: Each lot of this antibody is quality control tested by immunofluorescent staining with flow cytometric analysis.

Animals and other organisms

Policy information about studies involving animals; ARRIVE guidelines recommended for reporting animal research

Laboratory animals

The neuroblastoma O-PDX was propagated in one female nude adult athymic Foxn1-null mouse (Charles River Laboratories, strain code 553) via para-adrenal injection. At the time of injection, the mice are 6-8 weeks in age, and it takes 4-8 weeks for the O-PDX to grow.

Wild animals

This study did not involve wild animals.

Field-collected samples

This study did not involve field-collected samples.

Ethics oversight

Animal use was restricted to 1 female nude athymic mouse for para-adrenal injection of O-PDX cells. This study was carried out in strict accordance with the recommendations in the Guide to Care and Use of Laboratory Animals of the National Institute of Health. The protocol was approved by the Institutional Animal Care and Use Committee at St. Jude Children's Research Hospital. All efforts were made to minimize suffering. All mice were housed in accordance with approved IACUC protocols. Animals were housed on a 12-12 light cycle (light on 6 am and off 6 pm) and provided food and water ad libitum. Athymic nude female mice were purchased from Charles River Laboratories (strain code 553).

Note that full information on the approval of the study protocol must also be provided in the manuscript.

Human research participants

Policy information about studies involving human research participants

Population characteristics

This research was not designed as a population study. Only a small number of samples (2-7) are profiled and analyzed per cancer type. Most samples are from adults, with the remaining samples being pediatric (pediatric high-grade glioma and neuroblastoma).

Recruitment

Patients were not actively recruited for this secondary-use study. Instead, patients were recruited under the initial IRB protocols approved by our collaborating institutions (see "Ethics oversight" section). External sample cohorts were then added to the Broad's Molecular Classification of Cancer protocol (15-370B) and reviewed and approved by the Dana Farber Cancer Institute (DFCI) IRB. Patient population compositions are not expected to impact our results as our analyses were done on a per sample basis, rather than on patient populations.

Ethics oversight

Ethics oversight for the Molecular Classification of Cancer protocol (15-370B) is performed by the DFCI IRB. Samples added to this protocol also underwent IRB review and approval at the institutions where the samples were originally collected. Specifically, Dana-Farber Cancer Institute IRB approved the following protocols: lung cancer (IRB protocol 98-063), metastatic breast cancer (IRB protocol 05-246), neuroblastoma (IRB protocols 11-104 and 17-104), ovarian cancer (IRB protocol 02-051), melanoma (IRB protocol 11-104), sarcoma (IRB protocol 17-104), GBM(IRB protocol 10-417), and chronic lymphocytic leukemia (IRB protocol 99-224), and the St. Jude Children's Research Hospital IRB approved the following protocol: pediatric high-grade

glioma (IRB protocol 97BANK).

The XPD 09-234 MAST (Molecular Analysis of Solid Tumor) protocol for creating the neuroblastoma O-PDX sample was reviewed and approved by the St. Jude Children's Research Hospital IRB.

Note that full information on the approval of the study protocol must also be provided in the manuscript.

Flow Cytometry

Plots

Confirm that:

☒ The axis labels state the marker and fluorochrome used (e.g. CD4-FITC).

☒ The axis scales are clearly visible. Include numbers along axes only for bottom left plot of group (a 'group' is an analysis of identical markers).

☒ All plots are contour plots with outliers or pseudocolor plots.

☒ A numerical value for number of cells or percentage (with statistics) is provided.

Methodology

Sample preparation	For flow cytometry analysis of CD45+ depletion in the ovarian cancer ascites sample, cells were resuspended in PBS complemented with 2% fetal bovine serum and stained with FITC anti-human CD45 antibody (BioLegend #304006CD45, 1:200 dilution), PE anti-human EPCAM antibody (Miltenyi Biotech #130-113-264, 1:50 dilution), APC anti-human CD14 (BioLegend #367118, clone 63D3, 1:20 dilution), and PE-cy7 anti-human CD24 (BioLegend #311120, clone ML5, 1:20 dilution) for 20 minutes, and with 7-AAD (Invitrogen #A1310, 1:200 dilution) for 5 minutes. The same cells were also used for single-stain and unstained controls in order to perform compensation and adjust gating.
Instrument	BD LSRFortessa Cell Analyzer (Cat. No. 647177)
Software	BD FACSDiva Software Version 8.0.1; plots were generated with FlowJo Version 10.5.3
Cell population abundance	We used a CD45+ depletion strategy to prepare an ovarian ascites sample for scRNA-Seq. To assess how well our CD45+ depletion strategy worked, we took a sample of these prepared cells, with and without the CD45+ depletion, and performed flow cytometry. CD45- cells were enriched from 0.75% to 29.4% of the population, as determined using the anti-CD45 antibody. EpCAM+ cells were enriched from 0.17% to 4.9%, as determined by the PE anti-human EPCAM antibody.
Gating strategy	Cells were gated by FSC and SSC (35% of events retained for no depletion, 23% of events retained for depletion of CD45+ cells), doublets removed using FSC-A and FSC-H (100% singlets for no depletion, 99.8% singlets for depletion of CD45+ cells), live cells identified using 7-AAD (84.7% of cells retained for no depletion are live, 96.6% of cells retained for depletion of CD45+ cells are live), the distribution of immune and non-immune cells quantified using the CD45 antibody (99.3% of cells retained for no depletion are CD45+, 70.5% of cells retained for depletion of CD45+ cells are CD45+), and the distribution of EPCAM+ cells quantified using the EPCAM antibody (0.17% of the cells retained for no depletion are EPCAM+, 4.92% of cells retained for depletion of CD45+ cells are EPCAM+).

☒ Tick this box to confirm that a figure exemplifying the gating strategy is provided in the Supplementary Information.
